# RNAi-induced knockdown of *white* gene in the southern green stink bug (*Nezara viridula* L.)

**DOI:** 10.1038/s41598-022-14620-0

**Published:** 2022-06-21

**Authors:** Dariane Souza, Shawn A. Christensen, Ke Wu, Lyle Buss, Kaylin Kleckner, Constance Darrisaw, Paul D. Shirk, Blair D. Siegfried

**Affiliations:** 1 Entomology and Nematology Department, University of Florida, Gainesville, 32611 USA; 2 USDA-ARS Center for Medical, Agricultural and Veterinary Entomology, Gainesville, 32608 USA; 3 Syngenta Crop Protection AG, WST-540.1.17 Schaffhauserstrasse, 4332 Stein, Switzerland

**Keywords:** RNAi, Genetics, Genetic markers

## Abstract

The southern green stink bug (SGSB) *Nezara viridula* L. is one of the most common stink bug species in the United States and can cause significant yield loss in a variety of crops. A suitable marker for the assessment of gene-editing tools in SGSB has yet to be characterized. The *white* gene, first documented in *Drosophila*, has been a useful target to assess the efficiency of introduced mutations in many species as it controls pigmentation processes and mutants display readily identifiable phenotypes. In this study we used the RNAi technique to investigate functions and phenotypes associated with the *white* ortholog in the SGSB and to validate *white* as a marker for genetic transformation in this species. This study revealed that *white* may be a suitable marker for germline transformation in the SGSB as *white* transcript knockdown was not lethal, did not impair embryo development and provided a distinguishable phenotype. Our results demonstrated that the *white* ortholog in SGSB is involved in the pathway for ommochrome synthesis and suggested additional functions of this gene such as in the integument composition, management of hemolymph compounds and riboflavin mobilization.

## Introduction

The *white* gene was the first X-linked gene characterized in *Drosophila* that helped to identify the role chromosomes play in heredity^1–3^ and has been used for more than a century as a pleiotropic model to study a number of regulatory mechanisms in eukaryotes^4–14^.

In *Drosophila*, the protein subunit encoded by *white* is needed to form functional ATP-binding cassette (ABC) transporters most commonly known for moving ommochrome and pteridine pigment precursors across membranes^8,15–20^ and for controlling the appearance of pigments in compound eyes, ocelli, testes and Malpighian tubules^4,5,15,21^. The lack of pigmentation particularly in the compound eyes of *Drosophila*, has been a readily identifiable phenotype of *white* mutants that drives a general interest in characterizing orthologous sequences of this gene as a marker for genetic transformation across different insect taxa^22–33^. Stink bugs (Hemiptera:Pentatomidae) represent an increasingly important pest complex in which a marker for genetic transformation has yet to be characterized.

Most stink bugs are polyphagous^34,35^ and can cause significant yield loss in a variety of crops^36–48^. The introduction of transgenic crops targeting primary pests has been increasing the incidence of several stink bug species across the southern United States where the southern green stink bug (SGSB) *Nezara viridula* L. is considered one of the most common and primary stink bug pest^35,49–51^. Documentation of gene-editing systems in SGSB would open multiple avenues for continued research related to stink bug management^52^ and the *white* ortholog in this species may be a useful target to assess the efficiency of introduced mutations. Nevertheless, the function and phenotypes associated with the *white* ortholog in SGSB have never been documented and some previous investigations targeting *white* orthologs of hemipteran species suggest a diverse and contrasting capacity of using this gene as a marker for genetic transformation.

RNA interference (RNAi) experiments performed with the brown planthopper, *Nilaparvata lugens* (Stål), revealed that knockdown of *white* ortholog transcripts caused a significant reduction of ommochrome and pteridine pigments in their compound eyes and ocelli^30,53^ and was a reliable marker to confirm germline transformation^28^. By comparison, knockdown of *white* transcripts in the tarnished plant bug *Lygus hesperus* Knight, caused a whole body color depletion and some unexpected negative effects, such as lethargy, reduced body size, and softer and stickier cuticles indicating that *white* would be a less suitable marker for genetic transformation in this species^31^. A gene-editing approach targeting the *white* ortholog in the silverleaf whitefly *Bemisia tabaci* (Gennadius) showed that this marker allowed easy screening of *white* mutant nymphs and adults as they developed distinct eye colors relative to wild type, and allowed an investigation of the heritability of mutations generated^33^. More recently, RNAi and gene editing investigations performed with the milkweed bug, *Oncopeltus fasciatus* (Dallas), revealed that homozygous *white* mutations caused translucent patches in the integument, loss of pigments throughout the body, but were lethal during embryo development^32^. Embryonic lethality caused by the disruption of the *white* orthologues is not common among insect species but has been suggested in Lepidoptera^27^ and Hemiptera^54^, which is a major limitation of its use to document germline transformation.

Validation of gene editing markers is a necessary initial step to rule out lethality and unwanted deleterious phenotypes. RNAi is a gene silencing mechanism that is commonly used to investigate gene function and has been successfully triggered in stink bug species through microinjections of gene-specific double-stranded RNA (dsRNA)^55–57^ including in the SGSB^58,59^. Therefore, in our study we performed dsRNA microinjections targeting the *white* ortholog in SGSB aiming (1) to provide an initial evaluation of the phenotypes associated with the knockdown of this gene, and (2) to validate *white* as a marker for genetic transformation in the SGSB.

## Methods

### Insects

A reference SGSB population was established in the Insect Resistance lab, Entomology and Nematology Department, University of Florida, Gainesville, FL from a lab strain initiated from field collections that had been under laboratory rearing for many generations^60^. Insects were maintained on snap peas, sweet corn, peanuts, and provided with water through moisturized cotton in environmental chambers at 25 ± 1 °C, 65% RH and 14 light: 10 dark h photoperiod. Food and water were replaced twice a week.

### RT-qPCR primer efficiency test

SGSB transcriptome sequences of three target genes (*white*, *vermilion*, and *cinnabar*) known to be involved in eye pigmentation of *D. melanogaster*^15^ and of four candidate SGSB reference genes (*β-Actin*, *α-Tubulin*, *EF1-α*, and *GAPDH* ) were generously provided by Syngenta Ltd., Jealott’s Hill International Research Centre, Bracknell, Berkshire, UK. Identities of sequences were first verified by similarity search in BLASTx against the brown marmorated stink bug *Halyomorpha halys* (Stål) database (NCBI taxid:286706) (Supplementary Table S1) and later confirmed by the sequencing of SGSB-derived PCR products. Additionally, a phylogenetic tree was constructed in Geneious prime 2022.1.1. for white, scarlet and brown amino acid sequences that have been previously annotated in genomic studies and/or were found in BLAST results for the SGSB sequence targeted in this study, which confirmed the *white* identity (Supplementary Figure S1). Primers for target and reference genes (Supplementary Table S2) were designed using PrimerQuest® Tool^61^ and obtained from Integrated DNA Technologies, IDT (Coralville, IA). Total RNA was extracted from three SGSB pooled samples (12 eggs, two second instar nymphs and legs of two adults) using a Qiagen RNeasy mini kit (Germantown, MD, Cat. No. 74104) following manufacturer instructions. RNA quality and quantity were verified by spectrophotometry using the NanoDrop ND-1000 spectrophotometer (Thermo Scientific™, Waltham, MA). cDNAs were synthesized from 500 ng of total RNA using the Cloned AMV First Strand Synthesis kit (Invitrogen™ Life Technologies™, Carlsbad, CA, Cat. No. 12328-032). Samples were diluted tenfold and evenly mixed to obtain a single representative SGSB cDNA sample. The efficiencies of primers designed for target and reference genes of the SGSB were estimated from a fourfold serial dilution series of the pooled cDNA sample. Quantitative Real Time PCR (RT-qPCR) reactions were performed in a 20 μL final reaction volume containing 10 μL of SsoAdvanced™ universal SYBR® Green supermix (Bio-Rad, Hercules, CA, Cat No.172-5271), 1 μL cDNA, 0.3 μM of each primer and complemented with nuclease-free water. Reactions were carried out in triplicates in a CFX96™ Real-Time System (Bio-Rad Laboratories, Singapore) using hard-shell 96 well plates (Bio-Rad Laboratories, Hercules, CA, Cat. No. HSP9601). Temperature profiles included an initial heating step at 95 °C for 2 min, followed by 40 cycles of denaturation at 95 °C for 5 s and annealing at 60 °C for 30 s, one additional denaturation step at 95 °C for 5 s, and a final melting cycle from 65 to 95 °C (0.5 °C increments) for 5 s. A standard curve was generated for each primer pair and the regression correlation coefficient (R^2^) and PCR efficiency (E) for each standard curve were calculated^62^.

### Double stranded RNA (dsRNA) preparation

SGSB genomic DNA was extracted from one SGSB adult leg using a Qiagen DNeasy kit (Germantown, MD, Cat No. 69504) according to the manufacturer’s instructions. Polymerase chain reactions (PCR) were performed in a 50 μL final reaction volume containing 10 ng of template DNA, 1 × PCR Buffer (Tris pH 9.2, 16 mM ammonium sulfate, 1.75 mM MgCl_2_), 0.35 mM dNTP, 1 unit of Taq Polymerase (Thermo Fisher Scientific™, Vilnius, LT, Cat. No. EP0402), 0.2 μM of the forward (5′-AATGATAATGTCTTACCTCTAGACC-3′) and reverse (5′- TTTAGAATGTGCTTCCTACCG-3′) primers to amplify a 191 bp SGSB *white* exonal fragment. The amplified PCR product was analyzed by 1% agarose gel electrophoresis, purified using the GeneJET Gel Extraction Kit (Thermo Fisher Scientific™, Vilnius, LT, Cat. No. K0691) and confirmed by sequencing (Genewiz, South Plainfield, NJ). A T7 promoter was linked to the purified *white* DNA fragment by PCR using the gene-specific primers with the T7 sequence (5′-TAATACGACTCACTATAGGGAG-3′) appended to the 5′ end of each primer. Similarly, a non-specific green fluorescence protein (GFP) gene fragment of 503 bp was amplified from pGLO™ plasmid (Bio-Rad, Hercules, CA, Cat. No. 1660405EDU) by PCR using the forward (5′-T7- AGGTGATGCTACATACGGAAAG-3′) and reverse (5′-T7-ACAGGTAATGGTTGTCTGGTAAA-3′) primers. The PCR reactions were carried out using a C1000 Touch™ Thermal Cycler (Bio-Rad Laboratories, Singapore) and the temperature profiles included an initial heating step at 94 °C for 2 min, followed by 39 cycles of denaturation at 94 °C for 15 s, annealing at 60 °C for 30 s, and extension at 68 °C for 30 s. PCR products generated were then used for dsRNA synthesis using MEGAscript™ RNAi Kit (Invitrogen™ by Thermo Fischer Scientific™, Vilnius, LT, Cat. No. AM1626) according to manufacturer instructions (https://assets.thermofisher.com/TFS-Assets/LSG/manuals/cms_072987.pdf). The quality and quantity of purified dsRNAs were verified by NanoDrop.

### RNAi experiments

#### Selection of reference genes

A preliminary test was performed to verify the stability of candidate reference genes during RNAi-based microinjection experiments in SGSB. Two doses of *white* and *GFP* dsRNAs (150 and 750 ng) were injected in the abdomen of newly emerged adult SGSB females in a final volume of 3 µL using glass microneedles attached to a pneumatic microinjector (Narishige model IM-11-2, Tokyo, JP). Solutions were prepared in the MEGAscript™ elution buffer. Females injected with elution buffer-only were included as negative controls. A 1% v/v green food dye (McCormick & Co, Hunt Valley, MD) was added to all solutions to facilitate visualization of injections. Insects were immobilized on ice for about 5 min before injections. Injected females were maintained separated by treatment under SGSB rearing conditions in plastic cups (9.5 D × 7.5 H cm) covered with tulle fabric. Total RNA was extracted from one middle leg of each treated female 4 days after treatment (4 DAT) using the Qiagen RNeasy Mini Kit and quantified using a NanoDrop spectrophotometer. cDNAs were synthesized using the High-Capacity cDNA reverse transcription kit (Applied Biosystems by Thermo Fisher Scientific™, Vilnius, LT, Cat. No. 4368813) from 70 ng of RNA, following the manufacturer’s instructions. cDNA of six individual females from each treatment were used as biological replicates to verify expression levels of *white* and reference genes by RT-qPCR. The four candidate reference genes (*β-Actin*, *α-Tubulin*, *EF1-α*, and *GAPDH*) were tested during RT-qPCR analysis. RT-qPCR reactions were carried out as previously described (subsection “RT-qPCR primer efficiency test”). The stability of reference genes was examined by BestKeeper^63^, NormFinder^64^, GeNorm^65^, and the comparative ΔCt method^66^ on a web-based platform RefFinder (https://www.heartcure.com.au/reffinder/?type=reference). The *white* relative gene expression was calculated following Pfaffl^67^ using a geometric normalization factor estimated for multiple reference genes^65^. The statistical analysis was performed using a generalized mixed model in SAS 9.4 software^68^ with a completely randomized experimental design. Multiple comparisons of treatment means were performed using Fisher’s least significant difference procedure at significance level α = 0.05.

#### Knockdown of *white* transcript

RNAi experiments were conducted with SGSB to verify *white* knockdown efficacy overtime and corresponding phenotypic responses. Abdominal microinjections of *white* and *GFP* dsRNA (150 ng) were performed in each sex of newly emerged adults (< 24 h-old) and fifth instar nymphs. Nymphs were injected either less than 3 days prior to adult emergence (< 3 DPE) or more than 4 days prior to emergence (4 < DPE). Insects treated with elution buffer-only were used as negative controls. Newly emerged adults received 3 μL solution (50 ng dsRNA/μL) while nymphs received 2 μL (75 ng dsRNA/μL). Treated insects were maintained in plastic cups using standard rearing conditions. Food and water were replaced twice a week. Phenotypic changes were monitored and photographed using either a Canon EOS 7D digital camera and MP-E 65 mm lens or a Canon EF 100 mm f/2.8 Macro USM Lens at the Insect ID Lab at the University of Florida. Total RNA was extracted from one leg of each treated adult at 4, 10 and 30 days after treatment (DAT). For treated nymphs, total RNA was extracted after molting into adults at 2, 10 and 30 days after emergence (DAE). RNA extractions, cDNA syntheses, RT-qPCR reactions and *white* relative gene expression analyses were performed as previously described in the methods section. cDNA of four individual insects from each sex, treatment and time-point combination were used as biological replicates. Two reference genes (*β-Actin* and *α-Tubulin*) were used for the data normalization. The statistical analyses of *white* relative expression were performed using a generalized mixed model in SAS 9.4 software with a completely randomized experimental design. Sex, treatment, and time-points were adopted as fixed factors in the statistical model (2 × 3 × 3 and 2 × 4 × 3 factorial treatment designs for adults injected at < 24 h-old or during the nymphal stage, respectively). Multiple comparisons of treatment means were performed using Fisher’s least significant difference procedure at significance level α = 0.05.

#### Adult mortality and parental RNAi effect

Newly emerged adults of each sex of SGSB were treated following the methods described above (subsection “Knockdown of white transcript”). Nine to ten replicates of each treatment (150 ng *white* or *GFP* dsRNA, and elution buffer control) were performed. Treatment replicates were evenly represented by groups of six to ten insects. Treated insects were kept individually in small polystyrene boxes (5.9 L × 5.9 W × 7.8 H cm, Althor Products, Windsor Locks, CT, Cat. No. SM-1207) provided with food and water and covered with tulle fabric. Approximately 10 days after treatment, three untreated insects of the opposite sex were transferred into each box to allow mating. Adult mortality, egg laying, and phenotypic changes in progeny were monitored daily. To estimate the lethality of *white* knockdown in adult SGSB, the proportion mortality obtained from each treatment replicate at two different time-points (10 and 23DAT) were analyzed with a Beta-binomial distribution^69,70^ using a logit link function with a generalized mixed model in SAS 9.4 software. Multiple comparisons of treatment means were performed using Fisher’s least significant difference procedure at significance level α = 0.05. A completely randomized experimental design was adopted with a 2 × 3 factorial treatment design. Sex and treatments were adopted as fixed factors within each time point in the statistical model.

Collected eggs were transferred to Petri dishes (35 D × 10 H mm, Thermo Fisher Scientific, Roskilde, DK, Cat. No. 153066) and maintained in a growth chamber at 25 ± 1 °C, 65% RH and 14 light: 10 dark h photoperiod. Phenotypic changes of eggs and enclosed nymphs were monitored and recorded using a Canon EOS 7D digital camera and MP-E 65 mm lens at the Insect ID Lab at the University of Florida. Three eggs were detached from each egg cluster at the time of collection and stored at − 80 °C for subsequent RNA extraction to quantify *white* relative expression. Similarly, < 12 h-old first instar nymphs were either used for RNA extraction or maintained under rearing conditions. Total RNA was extracted from pooled samples of three < 24 h-laid eggs using the RNAqueous™-Micro Kit (Invitrogen™ by Thermo Fischer Scientific™, Vilnius, LT, Cat. No. AM1931) and from pooled samples of two first instar nymphs using the Qiagen RNeasy Mini Kit following manufacturer instructions. cDNA syntheses, RT-qPCR reactions and *white* relative gene expression analyses were performed as described in subsection 2.4.1. Three cDNA egg samples and four nymph cDNA samples originating from adult treatments were used as biological replicates of RT-qPCR reactions. Two reference genes (*α-Tubulin* and *EF1-α*) were used for the geometric normalization of *white* relative expression.

### Expression of ommochrome pathway genes

cDNA of SGSB adults at 30 days after emergence (30 DAE) that had been treated with *white* dsRNA either as newly emerged adults or prior to adult emergence (subsection “Knockdown of white transcript”) and pooled cDNA samples from first instar nymphs (< 12 h-old) from *white* dsRNA-treated mothers (subsection “Adult mortality and parental RNAi effect”) were used to measure the relative expression of two genes (*vermilion* and *cinnabar*) known to be involved in the metabolic pathway for the conversion of tryptophan to ommochromes in *Drosophila*^15^. These genes were assessed for potential differential regulation as an additional mechanism to prevent ommochrome precursor accumulation in the SGSB. Four adults from each sex and treatment combination and three pooled cDNA samples from nymphs were used as biological replicates. cDNA from buffer- and *GFP* dsRNA-injected treatments were used as wild-type controls. RT-qPCR reactions and relative expression analyses were performed as described in subsection “Selection of reference genes”. using two reference genes (*β-Actin* and *α-Tubulin*) for data normalization. The statistical analyses of gene relative expression were performed using a generalized mixed model in SAS 9.4 software with a completely randomized experimental design. Sex and treatments were adopted as fixed factors in the statistical model of the adult test (2 × 4 factorial treatment design), while treatment was the only fixed factor in the model testing first instar nymphs (unsexed). Multiple comparisons of treatment means were performed using Fisher’s least significant difference procedure at significance level α = 0.05.

### Metabolomic analysis of ommochrome biosynthesis

Ommochrome precursors (tryptophan, kynurenine and 3-hydroxy-DL-kynurenine) and riboflavin were evaluated in SGSB samples collected from RNAi experiments. The metabolites were measured in adults at 30 days after emergence (30 DAE) and in first instar nymphs at < 12 h after hatching. Adults that had been treated with *white* dsRNA either as newly emerged adults or prior to adult emergence (subsection “Knockdown of white transcript”) were tested as separated groups. First instar nymphs tested were the progeny of SGSB females treated with *white* dsRNA at < 24 h after emergence. Wild-type insects were used as controls. Pooled samples of six adults (1:1 sex ratio) or 60 nymphs (unsexed) were used as biological replicates. Six biological replicates of 25 mg ground tissue were prepared for each treatment. Adult samples were prepared using a porcelain mortar and pestle while nymph samples were prepared with polypropylene disposable pestles in 1.5 mL centrifuge tubes. All samples were processed in liquid nitrogen and stored at -80 °C until analysis. Extractions were performed as previously described^71^. Ultra-high-performance liquid chromatography-high-resolution mass spectrometry (UHPLC-HRMS) was carried out on a Q Exactive mass spectrometer coupled to a Vanquish LC System (Thermo Fisher Scientific, Waltham, MA, USA) by reverse phase gradient elution using an ACE Excel 2 C18-PFP column (2.1 mm X 100 mm, 2 μm; part # EXL-1010-1002U) in full scan positive (injection volume 2 μL) and negative (injection volume 4 μL) ion modes. Identification was assigned to features by m/z (≤ 5 ppm) and retention time (< 0.2 min), using a method-specific metabolite library produced from pure standards previously analyzed using the above-mentioned chromatographic gradient. Analytical standards of L-Tryptophan (CAS: 73-22-3), L-kynurenine (CAS: 2922-83-0), 3-hydroxy-DL-kynurenine (CAS: 484-78-6) and riboflavin (CAS: 83-88-5) were purchased from Sigma-Aldrich Corp., St. Louis, MO (Cat No. T0254, K8625, H1771, PHR1054, respectively). The data were acquired, processed, normalized, filtered and metabolites identified using MZmine 2 software^72^ as previously described^71^.

## Results

### RT-qPCR primer efficiency and selection of reference genes

The PCR efficiency of all primers designed for target and reference genes of the SGSB is provided in Supplementary Table S2, and all exhibited appropriate efficiency. The estimated E values were all within a 92–100% range. Also, a consistently high linear correlation (> 99%) between the log of cDNA dilution factors and mean RT-qPCR Ct values was found for all primers tested. From the reference genes tested, *α-Tubulin*, *β-Actin*, and *EF1-α* transcripts were the most stably expressed across different dsRNA treatments (Supplementary Table S3). Consequently, these three transcripts were chosen as suitable reference options to be used for geometric mean normalization of *white* relative gene expression in subsequent RT-qPCR analyses.

Significant knockdown of the *white* transcript ($${F}_{\left(\mathrm{4,25}\right)}=35.37;p<0.0001)$$ was observed in SGSB adult females injected with 150 and 750 ng *white* dsRNA relative to *GFP* dsRNA- and elution buffer-treated females (Supplementary Figure S2). There was no significant difference in the levels of transcript knockdown between the two *white* dsRNA doses tested ($${t}_{25}=0.02;p=0.9826)$$ with both providing > 92% knockdown at 4 DAT. Therefore, further RNAi-based experiments were all conducted using 150 ng dsRNA.

### RNAi experiments

#### *Knockdown of* white *transcript*

**Figure 1:**
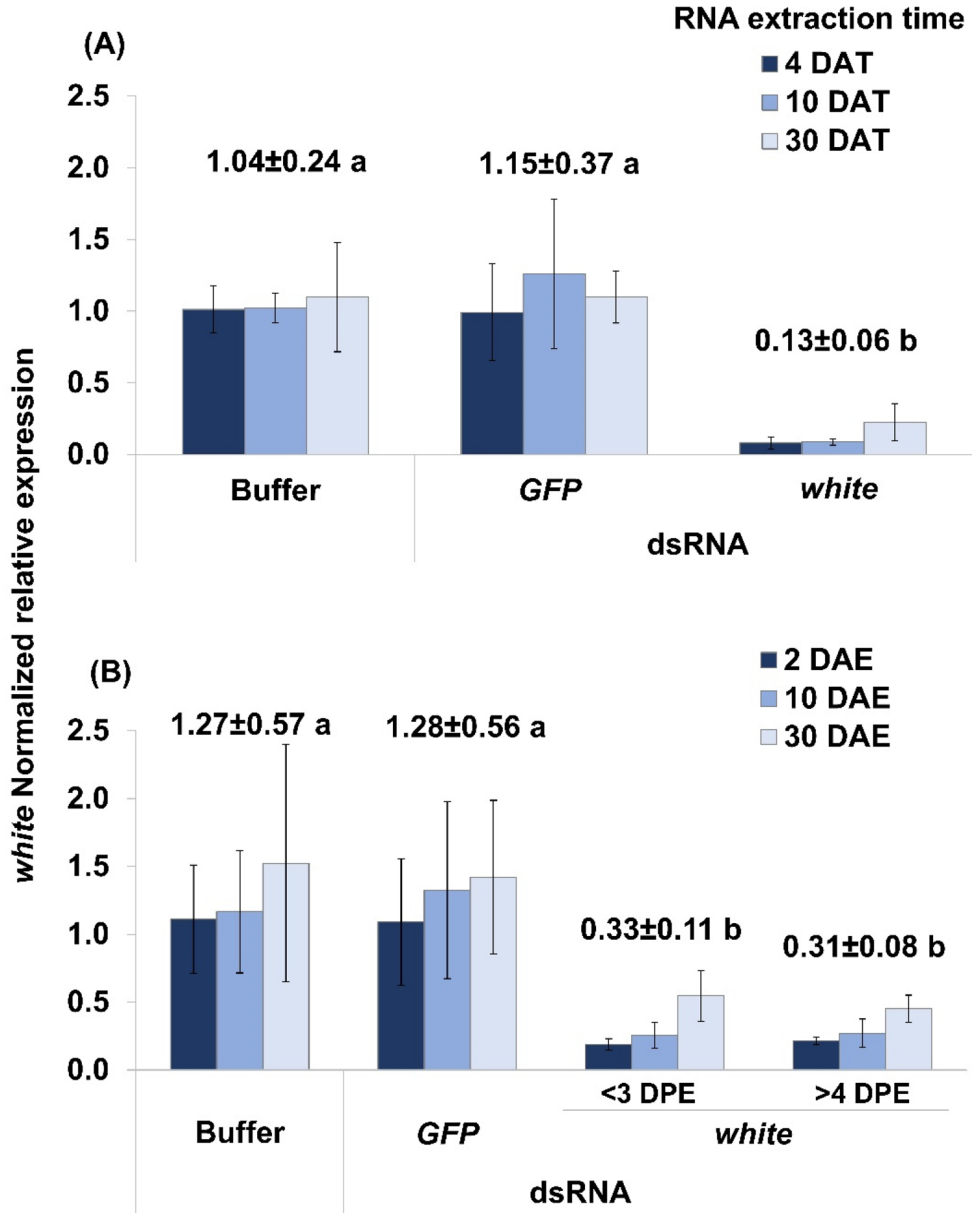
Transcript knockdown efficacy overtime of RNAi-based microinjection targeting the *white* gene of the SGSB. (**A**) SGSB adults had been treated at < 24 h after emergence. Significant *white* knockdown $({F}_{\left(\mathrm{2,69}\right)}=63.64;p<0.0001)$ was observed in adults treated with *white* dsRNA relative to *GFP* dsRNA- and buffer-treated controls at 4, 10 and 30 days after treatment (DAT). (**B**) SGSB adults had been treated during fifth nymphal stage at either less than three or more than 4 days prior to adult emergence (DPE). Significant *white* knockdown $({F}_{\left(\mathrm{3,92}\right)}=17.61;p<0.0001)$ was observed at 2, 10 and 30 days after adult emergence (DAE) in SGSB adults treated with *white* dsRNA either at < 3 DPE or > 4 DPE relative to *GFP* dsRNA- and buffer-treated controls. In each graph, bars discriminate *white* normalized relative expression means ± SE per time point and above bars show overall means ± SE per treatment across time. Treatment means followed by the same letter were not statistically different (Fisher’s LSD test, α = 0.05).

For knockdown experiments with newly emerged SGSB adults, there were no significant three-way interaction between sex, treatments and time points (${F}_{\left(\mathrm{4,54}\right)}=0.09;p=0.9843)$ or two-way interactions between sex and time (${F}_{\left(\mathrm{2,58}\right)}=0.65;p=0.5277$), and sex and treatment (${F}_{\left(\mathrm{2,58}\right)}=0.91;p=0.4077$) on the efficiency of *white* transcript knockdown. As there was no significant main effect of sex (${F}_{\left(\mathrm{1,62}\right)}=3.9;p=0.0529$), this factor was removed from the statistical model used. Similarly, time was removed from the model because there was no significant interaction effect between time and treatments (${F}_{(\mathrm{4,63})}=0.4;p=0.8067)$ or a main effect of time $({F}_{(\mathrm{2,67})}=1.11;p=0.3353)$ on the level of *white* transcript knockdown. Overall, a significant level of knockdown (~ 87%) $({F}_{\left(\mathrm{2,69}\right)}=63.64;p<0.0001)$ was observed at four, ten and 30 DAT in both sexes of SGSB adults treated with *white* dsRNA relative to *GFP* dsRNA- and buffer-treated controls (Fig. 1A).

For SGSB adults that had been treated during the fifth nymphal stage, there was no significant three-way interaction effect between sex, treatments and time (${F}_{\left(\mathrm{6,72}\right)}=0.24;p=0.9612)$ or two-way interaction effects between sex and time (${F}_{\left(\mathrm{2,78}\right)}=1.12;p=0.3315$), and sex and treatment (${F}_{\left(\mathrm{3,78}\right)}=0.74;p=0.5341$) on the efficacy of *white* transcript knockdown. The main effect of sex was also not significant (${F}_{\left(\mathrm{1,83}\right)}=0.01;p=0.9394$), so this factor was removed from the statistical model used. As there was no significant interaction effect between time and treatments (${F}_{(\mathrm{6,84})}=0.08;p=0.9977)$ and no significant main effect of time (${F}_{(\mathrm{2,90})}=2.34;p=0.1021)$ on the level of *white* transcript knockdown, this factor was also removed from the statistical model. Overall, a significant level of *white* transcript knockdown (~ 76%) $({F}_{\left(\mathrm{3,92}\right)}=17.61;p<0.0001)$ was observed at two, ten and 30 DAE in both sexes of SGSB adults treated during the fifth nymphal stage with *white* dsRNA either at < 3 DPE or > 4 DPE. This contrasts to no change in transcription for either the *GFP* dsRNA- or buffer-treated controls in all concurrent time points tested (Fig. 1B).

#### Adult phenotype

An observable change from wild-type green color to blueish green, as well as a more translucent integument was observed in 100% of both female (n = 150) and male (n = 150) SGSB adults treated with *white* dsRNA at < 24 h after emergence, which was not apparent in control treatments (Supplementary Figure S3). Changes in color patterns were apparent 10 DAT and were sustained throughout adult life. Fifth instar nymphs treated with *white* dsRNA (n = 300) molted into SGSB adults with distinct body colors as well that were sustained throughout adult life (Supplementary Figure S4).

**Figure 2:**
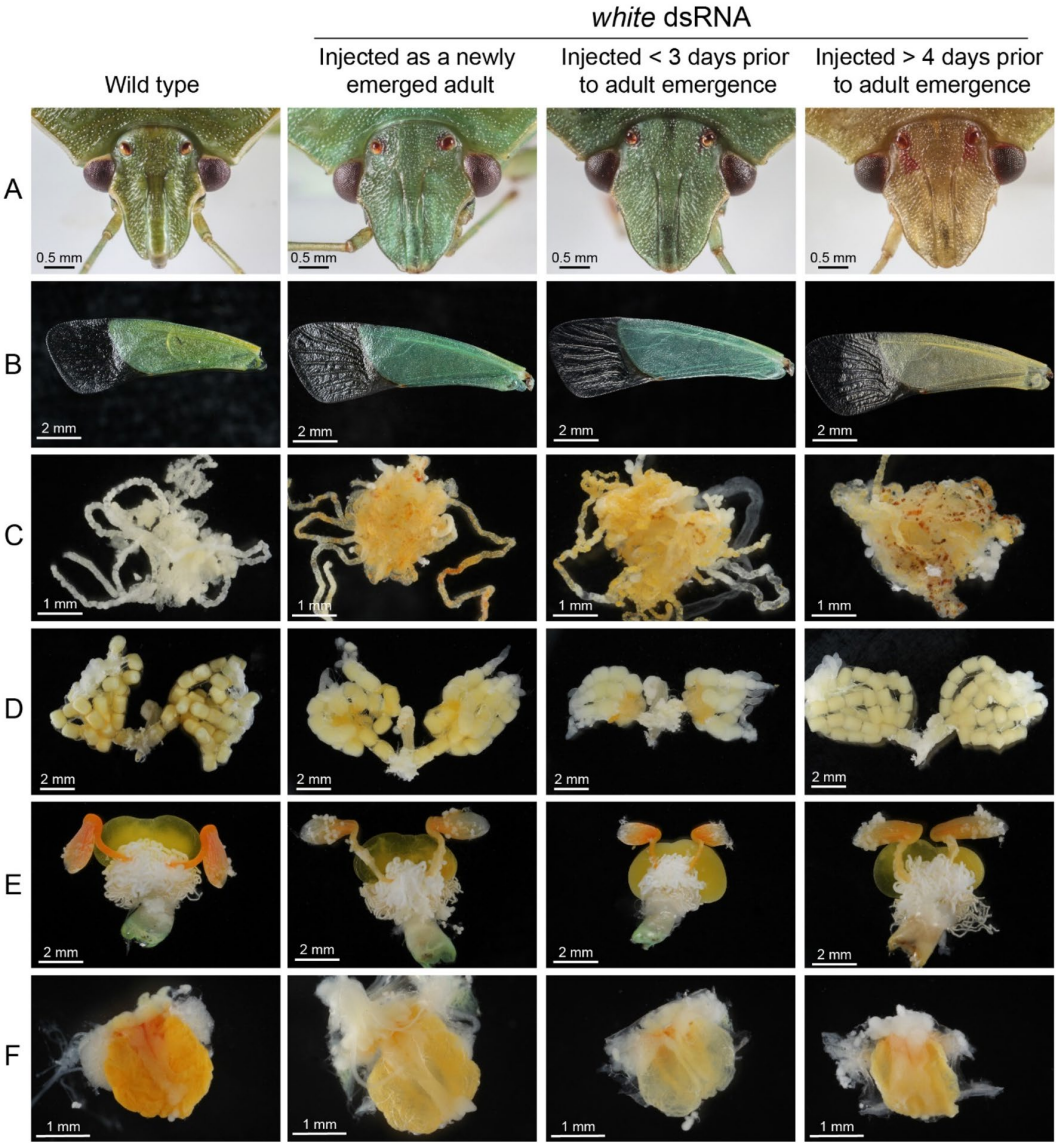
Phenotypes observed in 30 days-old SGSB adults following RNAi-based microinjections relative to wild type. Figure shows (**A**) head displaying compound eyes and ocelli; (**B**) forewing; (**C**) Malpighian tubules; (**D**) female gonads; (**E**) male gonads; and (**F**) metathoracic scent reservoir.

Male and female nymphs injected with *white* dsRNA at < 3 DPE (n = 150) developed a translucent integument as adults and a blue-green body color, which was paler than the blueish green phenotype observed in SGSB treated at the early adult stage. Nymphs that were treated with *white* dsRNA > 4 DPE (n = 150) developed a yellow color along with a more translucent integument as adults. The phenotype observed in *white* dsRNA-treated nymphs was distinct from the wild-type control treatments from the day of adult emergence until the end of adult life. Although a body color variegation was the most obvious SGSB adult phenotype generated by the *white* transcript knockdown, minor color differences in their compound eyes and ocelli were also observed (Fig. 2A). The predominant brown pigmentation of SGSB compound eyes and ocelli was reduced in all *white* dsRNA treated insects, exposing underlying red pigments. The red pigmentation was more obvious in SGSB adults treated at the nymphal stage > 4 DPE relative to the other treatments.

Dissections of mature SGSB adults (~ 30 days old) revealed that *white* transcript knockdown generated clear phenotypic differences of internal organs in this species (Fig. 2). Malpighian tubules that are normally transparent-white in wild-type SGSB adults were orange-yellow in all *white* dsRNA treated insects (Fig. 2C). Conversely, the orange-yellow color of gonads and metathoracic scent reservoir appeared discolored in SGSB adults that had been treated with *white* dsRNA (Fig. 2D–F).

#### Adult mortality

The mean proportion mortality observed in *white* dsRNA-treated adults was not significantly different from the mortality observed in *GFP* dsRNA- and elution buffer-treated control groups either by 10 DAT $({F}_{\left(\mathrm{2,53}\right)}=0.17;p=0.8421)$ or by 23 DAT $({F}_{\left(\mathrm{2,54}\right)}=1.47;p=0.2394)$ (Supplementary Figure S5A-B, respectively). Furthermore, there was no significant interaction effect between sex and treatments on the mortality of SGSB following RNAi-based microinjections at either 10 DAT $({F}_{\left(\mathrm{2,51}\right)}=0.57;p=0.5667)$ or 23 DAT $({F}_{\left(\mathrm{2,51}\right)}=1.89;p=0.161)$. However, there was a significant main effect of sex on SGSB adult mortality at 10 DAT, when males showed higher mortality across all treatments $({F}_{\left(\mathrm{1,53}\right)}=16.44;p=0.0002)$. No significant main effect of sex on SGSB adult mortality was observed at 23 DAT $({F}_{\left(\mathrm{1,53}\right)}=3.61;p=0.0629)$.

#### Parental RNAi

**Figure 3:**
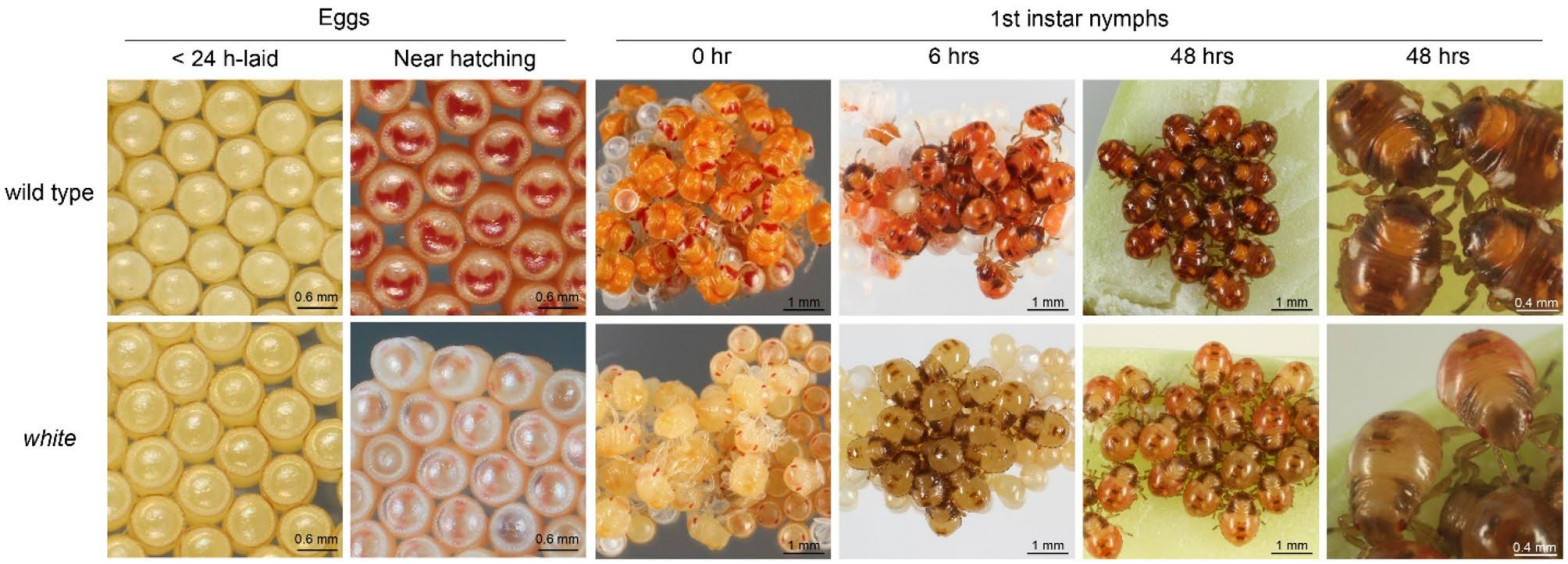
Phenotypes observed in the progeny of *white* dsRNA treated SGSB adult females relative to a wild type progeny of *GFP* dsRNA-treated females.

Newly laid eggs (< 24 h) from adult females treated with *white* dsRNA that were mated with non-treated males showed a significant knockdown of *white* transcripts (> 99%) $({F}_{\left(\mathrm{2,6}\right)}=9.1;p=0.0152)$ relative to eggs laid by *GFP* dsRNA- and buffer-treated female mating pairs (Supplementary Figure S6-B). However, there was no significant difference $({F}_{\left(\mathrm{2,6}\right)}=0.05;p=0.9558)$ of *white* relative expression in eggs laid by non-treated females that were mated with *white* dsRNA-treated males relative to control groups (Supplementary Figure S6-A). Phenotypic differences were detected in eggs from *white* dsRNA-treated females before hatching when embryos were more developed. Near hatching, 100% of eggs (> 500 eggs) collected from adult females treated with elution buffer or *GFP* dsRNA showed a typical wild-type phenotype, where a transparent chorion revealed an enclosed orange embryo with red compound eyes and a red crescent moon-shape forehead mark (Fig. 3). In contrast, 100% of eggs (> 500 eggs) collected from *white* dsRNA-treated females had very little color prior to hatching with only the red eyes and a paler red forehead mark of the embryo visually detectable through the egg chorion (Fig. 3). The phenotype of eggs produced by untreated females that were mated with *white* dsRNA-treated males were similar to wild-type eggs, suggesting that the response was maternally inherited.

All first instar nymphs (> 500 nymphs) generated from *white* dsRNA treated SGSB adult females also showed a distinct phenotype compared to the controls (Fig. 3). Upon hatching, nymphs from control groups displayed a wild-type bright orange integument and red compound eyes that darkened to brown by 48 h after hatching. Conversely, all first instar nymphs from *white* dsRNA treated mothers showed considerably reduced integumental color and predominance of red eye pigments-only up to 48 h after hatching (Fig. 3). Although there were clear phenotypic differences between first instar nymphs of *white* dsRNA-treated SGSB adult females and those of *GFP* dsRNA- and buffer-treated female controls, the *white* relative expression levels at < 12 h after hatching were not significantly different $({F}_{\left(\mathrm{2,9}\right)}=0.54;p=0.5999)$ amongst the different treatment groups (Supplementary Figure S6-C).

### Expression of ommochrome pathway genes

Significant downregulation of *vermilion* $({F}_{\left(\mathrm{3,28}\right)}=10.08;p<0.0001)$ was observed in SGSB adults that had been treated with *white* dsRNA relative to the wild type control, while the relative expression of *cinnabar* was not significantly different $({F}_{\left(\mathrm{3,28}\right)}=1.67;p=0.1959)$ among the same treatment groups (Supplementary Figure S7-A). There was no significant two-way interaction effect between sex and treatments on the relative expression of either *vermilion* (${F}_{\left(\mathrm{3,24}\right)}=0.24;p=0.8698$), or *cinnabar* (${F}_{\left(\mathrm{3,24}\right)}=1.03;p=0.3987$). The main effect of sex was also not significant for *vermilion* (${F}_{\left(\mathrm{1,27}\right)}=0.08;p=0.7750$), or *cinnabar* (${F}_{\left(\mathrm{1,27}\right)}=0.49;p=0.4884$) in SGSB adults, so this factor had been removed from the statistical model. First instar nymphs from *white* dsRNA-treated mothers did not show significant differences in the expression of either *vermilion* (${t}_{\left(4\right)}=0.3;p=0.7813$) or *cinnabar* (${t}_{\left(4\right)}=0.23;p=0.8283$) relative to wild-type controls (Supplementary Figure S7-B).

### Metabolomic analysis of ommochrome biosynthesis

**Figure 4:**
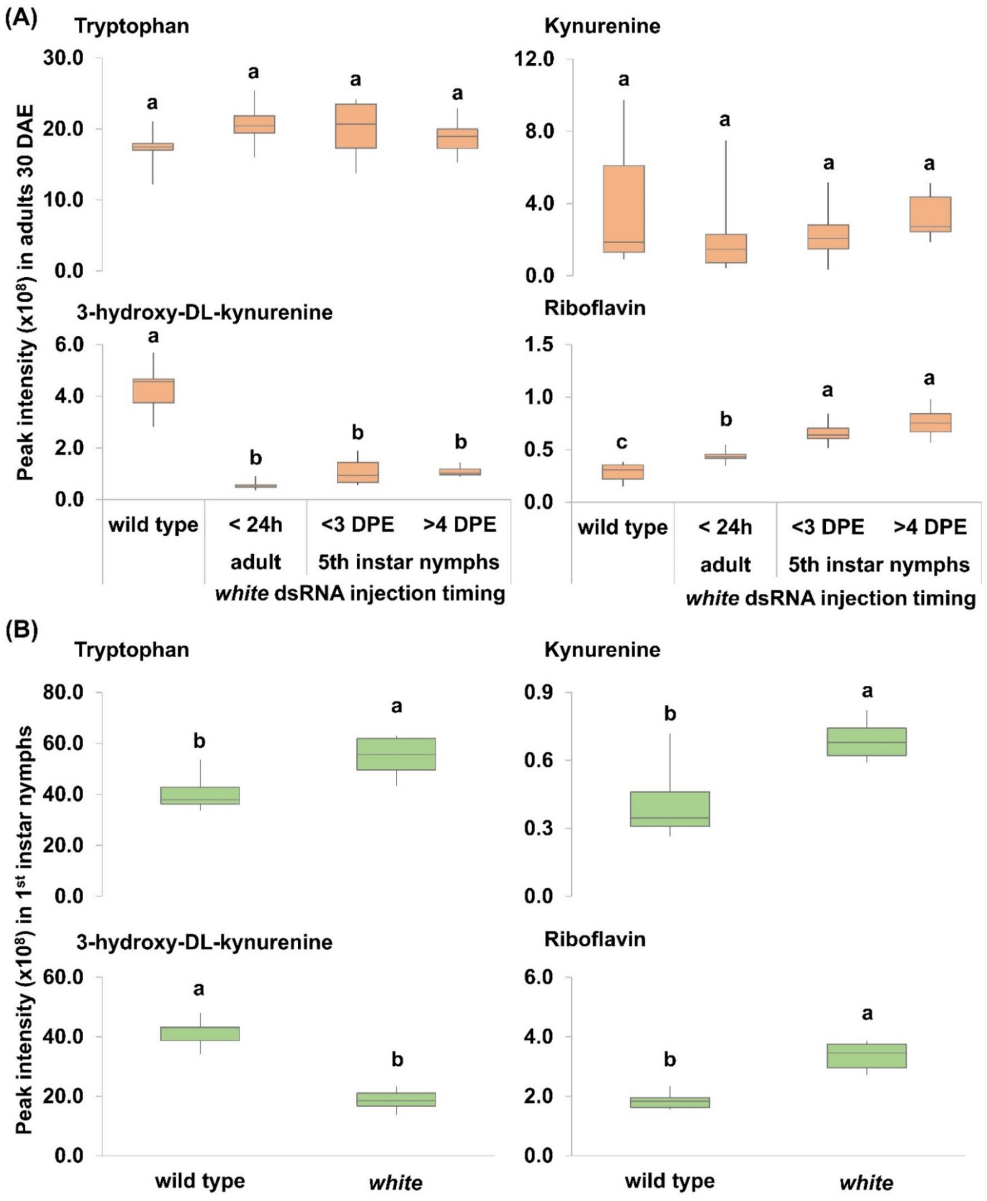
Metabolomic analysis of riboflavin and ommochrome biosynthesis in SGSB comparing *white* and wild type phenotypes observed during RNAi-based experiments. (**A**) adults at 30 days after emergence (DAE) that were treated with *white* dsRNA as < 24 h newly emerged adults or as nymphs either at < 3 days prior emergence (DPE) or > 4 DPE. Metabolites measured in adults were: tryptophan $({F}_{\left(\mathrm{3,20}\right)}=1.23;p=0.3244)$ ; kynurenine $({F}_{\left(\mathrm{3,20}\right)}=0.52;p=0.6754)$; 3-hydroxy-DL-kynurenine $({F}_{\left(\mathrm{3,20}\right)}=51.25;p<0.0001)$; and riboflavin $({F}_{\left(\mathrm{3,20}\right)}=23.08;p<0.0001)$. (**B**) first instar nymphs (< 12 h-old) originated from mothers treated with *white* dsRNA at < 24 h adult stage. Metabolites measured in first instar nymphs were: tryptophan $({t}_{\left(10\right)}=3.21;p=0.0093)$; kynurenine $({t}_{\left(10\right)}=3.51;p=0.0056)$; 3-hydroxy-DL-kynurenine $({t}_{\left(10\right)}=9.25;p<0.0001)$; and riboflavin $({t}_{\left(10\right)}=6.46;p<0.0001)$. Treatment means followed by the same letter were not statistically different (Fisher’s LSD test, α = 0.05).

Relative to wild type controls, SGSB adults that had been treated with *white* dsRNA generated significantly lower peak intensities of 3-hydroxy-DL-kynurenine $({F}_{\left(\mathrm{3,20}\right)}=51.25;p<0.0001)$ and significantly higher peak intensities of riboflavin $({F}_{\left(\mathrm{3,20}\right)}=23.08;p<0.0001)$, while tryptophan $({F}_{\left(\mathrm{3,20}\right)}=1.23;p=0.3244)$ and kynurenine $({F}_{\left(\mathrm{3,20}\right)}=0.52;p=0.6754)$ measurements were not significantly different (Fig. 4-A). Furthermore, riboflavin intensity was significantly higher in adults treated with *white* dsRNA during the fifth nymphal stage at < 3 DPE $({t}_{\left(20\right)}=3.43;p=0.0026)$ and > 4DPE $({t}_{\left(20\right)}=5.07;p<0.0001)$ than in adults treated at < 24 h after emergence (Fig. 4-A). First instar nymphs from SGSB females that had been treated with *white* dsRNA at < 24 h after emergence produced significantly higher peak intensities of tryptophan $({t}_{\left(10\right)}=3.21;p=0.0093)$, kynurenine $({t}_{\left(10\right)}=3.51;p=0.0056)$ and riboflavin $\left({t}_{\left(10\right)}=6.46;p<0.0001\right)$ and significantly lower peak intensity of 3-hydroxy-DL-kynurenine $({t}_{\left(10\right)}=9.25;p<0.0001)$ relative to the wild-type control (Fig. 4-B).

## Discussion

Microinjections of *white* dsRNAs into fifth instar nymphs and newly emerged SGSB adults effectively reduced *white* transcriptional levels throughout the SGSB adult life without causing lethality. Furthermore, a parental *white* knockdown effect was confirmed in SGSB by our RNAi experiments. Parental or transgenerational RNAi is the process of interfering with gene transcription in the progeny by treating parents with gene-specific dsRNAs. We found more than 99% knockdown of *white* transcripts in fertilized eggs of SGSB females treated with *white* dsRNAs, which significantly impacted the associated metabolism and phenotype of eclosed nymphs but did not prevent embryo development. Previous studies have demonstrated parental RNAi in Pentatomidae bugs such as the brown stink bug *Euschistus heros* (F.)^55^ and the brown marmorated stink bug *H. halys*^56^. Parental RNAi has also been investigated in a number of other heteropteran species such as kissing bugs^73,74^, water striders^75,76^ and milkweed bugs^32,77^ by treating females with dsRNAs. It should be noted that most parental RNAi cases documented for insects refers to a maternal effect where mothers were treated with dsRNAs. Little is known about paternal RNAi where fathers mediate the transgenerational response, and in some cases the words parental and paternal are incorrectly exchanged. Our results suggest that parental RNAi targeting the *white* transcription of SGSB was a maternal effect and may be achieved by delivering *white* dsRNAs into females only.

Gene expression knockdown allows the investigation of the targeted gene function and in our study, it revealed the physiological basis of the SGSB *white* in pigmentation of adults, embryos and first instar nymphs. At the aforementioned developmental stages, the wild-type colors of SGSB compound eyes, ocelli and integument were clearly disrupted after *white* transcript knockdown, and a subsequent metabolomics investigation confirmed an impact on the pathway for the conversion of tryptophan to ommochromes in this species. The *white* gene first discovered in *Drosophila* encodes a subunit of an ABC transporter recognized for loading pigment granules with pigment precursors such as 3-hydroxykynurenine^1,6,78,79^. The injection of *white* dsRNA into SGSB individuals significantly reduced the levels of 3-hydroxykynurenine supporting results reported for *D. melanogaster* Meigen^79,80^ and *Ephestia kühniella* (Zeller)^81^ where *white* null mutants exhibit reduced levels of this metabolite. Moreover, the progeny of SGSB females treated with *white* dsRNAs also showed reduced levels of 3-hydroxykynurenine confirming a persistent parental RNAi effect for the *white* gene.

The fact that 3-hydroxykynurenine is not properly transported across membranes of pigment granules in the absence of the *white* protein subunit^20,79^ could hypothetically cause a lethal accumulation of this compound in the insect hemolymph and/or cytosol^82–84^. It has been reported that insects carrying *white* mutations exhibit significantly reduced accumulation of 3-hydroxykynurenine in the body by an enhanced rate of excretion^22,79,83^. The current study examined genes involved in the upstream pathway for ommochrome synthesis during the conversion of tryptophan into N-formyl kynurenine (*vermilion*) and/or during the conversion of kynurenine into 3-hydroxykynurenine (*cinnabar*) and assessed potential differential regulation as an additional mechanism to prevent ommochrome precursor accumulation in the SGSB. Treatment with white dsRNAs resulted in significant downregulation of *vermilion* in SGSB adults while *cinnabar* expression was similar to untreated controls. However, in first instar progeny of females treated with *white* dsRNA where *white* transcript knockdown was achieved during embryo development but not after egg hatching, neither *vermilion* nor *cinnabar* were differentially expressed relative to controls. The levels of tryptophan and kynurenine in *white* dsRNA-treated adults did not differ from untreated controls while the levels of both metabolites were higher in first instar nymphs of *white* dsRNA treated females than in wild type nymphs. Previously, *Drosophila* mutants showed that the activity of *vermilion* encoded enzyme (tryptophan oxygenase) could be controlled by the concentration of kynurenine and that this enzyme is rate limiting in the catabolism of tryptophan^16^. Although the precise regulatory mechanisms remain uncertain, our results suggest that differential regulation of *vermillion* may be a SGSB response to *white* transcript knockdown that prevented tryptophan and kynurenine accumulation in adults. Furthermore, SGSB nymphs carrying the parental *white* RNAi effect could still maintain significantly low levels of 3-hydroxykynurenine regardless of the accumulation of its precursors tryptophan and kynurenine in their system, which suggests that regulation of other genes and/or an enhanced rate of excretion may prevent the 3-hydroxykynurenine accumulation in SGSB.

In *Drosophila*, the protein subunit encoded by *white* heterodimerizes with another subunit encoded by either *scarlet* or *brown* to form a functional ABC transporter for either ommochrome or pteridine pigment precursors, respectively^15,18–20^. Ommochromes are derived from tryptophan and are mostly brown, while pteridine pigments are derived from guanosine triphosphate and are characterized by yellow to red color variations^85–87^. Although we did not investigate pteridine related precursors and pigments in the SGSB, we found that *white* transcript knockdown in this species not only significantly reduced brown pigments in the eyes of adults and nymphs, but whole-body depletion of red-related pigments was apparent. Some wild populations of SGSB have been reported to carry genetic mutations conferring body color polymorphisms that range from the most frequent green morph to yellow and orange^88–90^. The color of SGSB adults is also known to change from green to russet during winter in high latitudes^91,92^. Furthermore, the pteridine erythropterin was detected in the integument of a closely related pentatomid stink bug species^93^. Thus, it is possible that pteridine pigments are present in the SGSB and that *white* may be involved in both ommochrome and pteridine pigment pathways in this species. Parental RNAi experiments performed on water striders where *white* transcription knockdown depleted all colors during embryo development confirmed that the *white* ortholog in these heteropterans is involved in both ommochrome and pteridine pathways^75^. It was further demonstrated in the same study that eye pigments of water striders are a combination of ommochromes and pteridines while body pigmentation was only associated with pteridines. Conversely, investigations on the blood-sucking heteropterans *Triatoma infestans* Klug and *Rhodnius prolixus* Stål suggest that only ommochrome pigments are present in the eyes of both wild-type and red-eyed mutants^94,95^. An investigation of pteridine precursors and derived pigments will be required to confirm the presence of this pigment pathway in SGSB and to characterize its spatial–temporal distribution across different tissues and life stages.

Some studies suggest that *white* may be involved not only in the transport of ommochrome and pteridine precursors but of other pigmented compounds, such as riboflavin, an orange-yellow B vitamin stored in the testes and Malpighian tubules of some insects^85,96–100^. Riboflavin has been associated with eye pigment pathways^4,21,85,99,101^. For example, the level of riboflavin was strongly reduced in testes of a white-eyed mutant strain of *E. kühniella*^99^ and *Anopheles gambiae* Giles^23^, and the testes and Malpighian tubules of white-eyed mutants of *Drosophila*^4,6,21,80^. More recently, investigations on mutant strains of the silkworm *Bombyx mori* L. showed that some mutations of the *white* and *brown* orthologs prevented an accumulation of riboflavin in Malpighian tubules^102,103^. The reproductive organs of more mature wild-type SGSB adults, such as testes and vas deferens of males and ovarioles of females are normally yellow or orange-yellow^104^. The metathoracic scent reservoir is another orange-yellow organ found in SGSB adults^105^. However, we observed that in adults that had been treated with *white* dsRNAs, especially earlier in the fifth nymphal stage, these organs were significantly discolored suggesting that the *white* encoded protein subunit may be involved in the transport of orange-yellow pigmented compounds into gonads and metathoracic scent reservoir of the SGSB. Dissections also revealed that SGSB adults that had been treated with *white* dsRNA developed orange-yellow Malpighian tubules instead of white-transparent ones normally found in wild-type adults.

Although we did not measure the pigmentation profile of SGSB organs individually to confirm that their differential pigmentation is due to riboflavin mobilization, we observed an overall accumulation of riboflavin in SGSB that had been treated with *white* dsRNAs and in their progeny suggesting that this compound was not being properly stored and/or catabolized relative to wild type controls. Bacterial symbionts are abundant in the hemolymph of hemipterans where they deliver essential amino acids and riboflavin to the hosts^106–110^. Thus, an impact on the transport of riboflavin could potentially result in the accumulation of this yellow compound in the hemolymph if not properly stored, and/or cause longer retention of this compound already deposited in specific organs, for example. It is possible that riboflavin is stored in the Malpighian tubules of SGSB during immature stages and then gradually transported to reproductive organs as they mature. Previous studies documented that in Lepidopteran species riboflavin content in Malpighian tubules and gonads are inversely correlated suggesting mobilization of this compound from one organ to the other^99,111^.

The relationship between *white* and riboflavin mobilization in the SGSB has yet to be clarified. Nevertheless, *white* has been implicated in the transport of cyclic GMP^9^, which is a signaling molecule involved in Malpighian tubule secretion in *Drosophila*^112,113^ and *R. prolixus*^114^*.* A microarray-based atlas of gene expression in multiple tissues in *Drosophila* named FlyAtlas (http://flyatlas.org)^115^ showed that *white* is most highly expressed in Malpighian tubules indicating a major local function of this gene. Interestingly, Malpighian tubules are responsible for regulating uric acid levels in different insect species^116^ and null *white* mutations were associated with transport defects of uric acid and its purine precursors in Diptera^4,117^ and Lepidoptera^118^. Reduced accumulation of uric acid in the epidermal cells of *Bombyx* and *Anopheles* larvae produced translucent skin and increased sensitivity to UV damage^117–120^. We found translucent skin in SGSB adults that had been treated with *white* dsRNAs, which could be an impairment of uric acid accumulation in their integument and will require further investigation.

The color of the adult hemolymph released during dissections was considerably different among the *white* SGSB phenotypes induced by RNAi treatments. SGSB adults that had been treated with *white* dsRNAs during the last nymphal stage developed a yellow integument and green hemolymph, while those treated near or after the adult molting period developed a blue-green integument, and discolored hemolymph similar to wild type SGSB adults. The green hemolymph of SGSB nymphs is made of a mixture of yellow and blue compounds^121^ and our results indicate that the *white* encoded protein subunit may be involved in their transport. Furthermore, some major physiological processes happening around 3 days prior to adult molting shifted the effect of *white* transcript knockdown on the integument greening of SGSB adults, turning it more blue than yellow. Perhaps an interaction between hormones released prior molting and pigmented compounds present in the hemolymph of SGSB nymphs that may be retained in the adult integument could have been impacted by *white* transcript knockdown. For example, copper-containing hemocyanins/hexamerins are blue proteins found in the hemolymph of many arthropods^122–124^ that are known to participate in the molting process^125–128^ and riboflavin transport^111^. Hexamerins with a blue cromophore (biliverdin) cause stage-dependent changes in the blue coloration of the hemolymph and fat body of the bean bug *Riptorus clavatus* (Thunberg) under the control of juvenile hormone^129,130^. Also, a hormone known to regulate red-related pigment transport in the integument and compound eyes of crustaceans has been previously detected in the corpora cardiaca of the SGSB^131,132^. Additional research is needed to characterize the origin and transport of integument pigments during SGSB adult molting and to clarify the involvement of *white* in the process.

This study revealed that the *white* ortholog in the SGSB is involved in the ommochrome pigment pathway and suggests additional functions of this gene such as riboflavin mobilization, management of hemolymph compounds and integument composition. Further investigations are needed for a complete characterization of this gene in the SGSB including verification of fitness cost and/or advantage associated with *white* transcript knockdown in this species. The fact that *white* transcript knockdown in the SGSB was not lethal or impaired embryo development while providing a readily identifiable phenotype may support the use of this gene as a marker for genetic transformation in this species, which would also allow additional investigations on the function and heritability of this gene.

## Supplementary Information

Supplementary Information.

## Data Availability

The datasets generated and /or analyzed during the current study are available from the corresponding author on reasonable request.
