# Supplementary material for: RNAi-induced knockdown of white gene in the southern green stink bug (Nezara viridula L.)
Source: Sci Rep. 2022 Jun 21;12:10396. doi: 10.1038/s41598-022-14620-0 (PMC9213411; doi:10.1038/s41598-022-14620-0)
Supplement: Supplementary file 1 — Supplementary Information. [file 41598_2022_14620_MOESM1_ESM.docx]

**Supplementary Information**

**Table S1.** SGSB cDNA sequences of target and reference genes investigated in this study.

| **Gene ortholog** | **Open reading frame (ORF)**  **SGSB cDNA sequence** | **BLASTx *H. halys* top hit (%query cover; %identity)** |
| --- | --- | --- |
| ***white*** | ATGACTATCCACGAAGAACACGAGCCTCTTCTTCAGGCAGCACCCAACGGCGGTTACCGGAGCACTTACAGAGGATCCAATAATGATAATGTCTTACCTCTAGACCCTGTCAAAAAACCTAAAGAAGGGGTTCAATTATATCCCACAGAAAGTCTCACTTACACTTGGTCTGGAATAAATGTCTTTGGTCGAGGAGAACCGAAGAAAAGAAAGATCATTAGTAAATTATTTTGCCGCAATACTGAGAGGTCCGGTAGGAAGCACATTCTAAAAAATGTTACGGGAATGGCTCTTCCTGGAGAACTCATGGCACTTATGGGATCTTCTGGTGCTGGAAAAACAACCTTACTAAATACACTAAACTTCAGGTCACCACCTTCTGTAGAAGTAACAGGACGGAGAGCCCTTAATGGAATTCCCGTGAATGCTAAAACTTTGAGCTCCCTTTCTGCCTACGTACAGCAGGATGATCTCTTCATAGGAACTCTCACAGTTCGAGAACATCTTATATTTCAGGCACTTGTGAGAATGGATCGACATATTCCATTTGAAACAAGAATGGCTCGAGTAGAAGAAGTTATATCCGAGTTAACTCTTAGTAGATGCCAAAATACCATCATCGGTGTTCCTGGAAAATTAAAGGGGATATCGGGAGGAGAAATGAAAAGGCTTTCTTTTGCTTCTGAGGTATTGACAGACCCTCCATTAATGTTTTGTGATGAGCCTACATCTGGATTAGATTCTTTTATGGCTCAGAATGTAGTAACTGTTTTGAAATCTCTTGCAGAAAAAGGAAAAACTGTAATCTGTACAATTCACCAACCTTCATCAGAGGTTTATGGTATGTTTGATAAAGTACTTTTAATGGCTGAAGGCAAAATGGCATTTTTAGGAACACCAACAGAAGCAAACGAGTTTTTTAAAACGATGGGCGCAGCATGTCCTAGTAACTATAACCCAGCAGATTTCTTTATACAATTGCTAGCTGTCGTTCCAACCCGTGAAGAAACATGCAGAAATATGATAGAAATGGTGTGCGATTCATATAAGAACTCAGACATCGGAGAGAAAATGATATTACAAGCAGAACTGGCAACGCAGGGCACAAAAAATAATTGGGGTATTTGGGCAGACAATTGGGCCTCGCCGAAGAATCTGTCGCCGTACAAAGCTTCCTGGACCGCTCAGTTCCGGGCAGTTCTTTGGAGGTCTTGGCTCAGTGTAATGAAGGAACCGGTACTCATCAAAGTCCGCATGCTCCAGACATTTATGGTTGCCCTTATGATTGGCGTGATATATTTTGGGCAGGAGCTCGATCAGGATGGTGTGATGAATATAAATGGTGCGCTATTCATCTGTCTCACAAATATGACATTTCAGAACGTCTTTGCTGTTATTAATGTATTCTGTTCTGAACTTCCTGTTTTCTTAAGAGAGCATATGAACGGGATGTATCGGACGGATATCTATTTCTTGTGTAAAACTTTGGCAGAGGTACCAGTATTTCTCGCAGTTCCACTTATTTTCACTTCGGTGATCTACTACATGGTTGGTCTTAACCCTGCGTTCGTCAGGTTTTTGTCGGCAGCACTCATTATAACTTTGGTCAGCAATGTTGCAACTTCCTTTGGGTACTTCATATCATGTGTCAGCTCAAGCATATCTGTGGCATTGTCTATTGGGCCTCCTGTTATAATTCCATTCTTACTGTTTGGAGGTTTCTTCCTCAATGCAGGATCCGTTCCACCATACTTCAAGTGGCTAAGCCAACTTTCTTGGTTCAAGTATGGCAATGAGGCTTTACTGATCAATCAATGGGAAGGCATAGAGACAATCTCTTGTACCAGGTCGAATACTACTTGTCCTAGGGATGGCCATGTAATTTTAGAAACTTACAATTTCAGTGAGGAAAATTATCTGGGTGATTACCTGGCTCTTACTGGCCTGATAGTTGGTTTCAGGGTACTTGCTTTCTTTGCGCTACTGAGCAAAACGTATTCTAAAAGAAGATAA | XP_014285221.1\| protein white-like, (99%; 97.01%) |
| ***vermilion*** | ATGGATACACAGCAGAGAATTGAAGCTGCTAAACTGACAGAATCTGAAAATGGAATGCTGTATGGCGAATATCTTCAATTAGACAAAATTTTATCAGCCCAACGGCTCTTAAGCGAAGTACACAAAGCCACTGTTCCCGATGAACACTTATTTATCATTACGCACCAAGCATACGAACTATGGTTCAAACAAATTATATTCGACCTGGATTGGATAATAGCAGTTTTCAGTAACCACATGGGTCTAGACGAAAGTGAAACATTAGGAATTATAAAGCGGCTCAATAGAATAGTTCTTATACTGAAGGTGTTAGTAGAACAAGTGATGGTTTTGGAAACGATGACCCCATTGGATTTTATGGACTTCCGAGGATATCTTTGTCCTGCATCTGGGTTTCAAAGCATGCAATTCAGAGACATCGAAAAAAAACTTGGACTTAGACTGGAAAATAAATCACTTTTAAAACCATGTGATCAAAATAAAGATAAAGAGATTCAAGAAAATTCAAAAGAAAATGATTTATCTTTGGCAGAATTAGTTGAGAGATGGTTAGAAAGGACTCCTGGTCTTGAAGAAAATGGATTTAACTTTTGGGGAAAGTATAAAAAAAATGTTGAATCTTTACTTAACATGTACTACAATGCAACACTGGATGCTAAGACAGATTGTGAAAAGCAAAAAAGAATGGCTGACTACGAGAGGAGAAAAGCTACCTTTGATTCAGTCACAGACATTAAACTACATGAATCCTTAGTTCAAAGAGGGGAAAGACGATTCAGGCACAAGGCTTTACAAGGAGCAATAATGATAACCTTTTATCGAGATGAGCCACGTTTCAGTCAGCCACACCAAATCCTTATGTTGCTTATGGACATAGACTCATTAGTAACAAAATGGAGATATAATCATGTTATAATGGTTCAAAGGATGATTGGATCTCAAAGTCTAGGAACTGGTGGATCTTCCGGTTATCACTACCTAAGATCAACATTAAGCGACCGCTATAAAGTTTTCTTGGATCTCTTCAATCTCTCTTCTTATTTACTTCCTCGAGGATACATACCACCATTGAGCAGTCAAATTAGAAGTCAAATTAATCTATATAAAGATTATATCAGCCAAACAAATGATGACACATCTGCATAA | XP_014281352.1\|tryptophan 2,3-dioxygenase, (99%; 94.49%) |
| ***cinnabar*** | ATGAAGGTATTAATAGTCGGAGGAGGTCTGGTCGGCTCGCTGTGCGCCTGTTATTTTGGTTTGCGAGGCCATGAAGTGCACATTTACGATTTTAGAAATGATCCTAGAACTGAAAAACTCTCAAAAGGACGATCCATCAACTTAGCACTTTCCACAAGAGGTATAGAAGCTCTCTCACGTGTTGGGGTCGAGGGAGTAATCTTGGAACATGGCATTCCCATGAAAGGCAGGATGATTCACTCGAAAAGTGGAGAGAAGAAGAGTATTCCCTATGATCCAGTATACAAGAAGTGTATATACTCAGTTGGAAGAAAACATTTGAACGAGTTACTCATCACAGAAGCCGAAAAGTATCAAAATGTTCATCTTCACTTCAATCATAAAATAACTTCCGTGAATTTCGAGGAGAAGAAAATATTCTTTAAATCGGCTGACAACAATAATTCTGAATTGAAAGAAGACAAAGGCGATCTAATCGTGGGTGCTGATGGGGCTTTCTCAATCGTGAGGCGAGAGATGATGAAGCAGCCCATGTTCAACTTCAGTCAGACTTACATCGAGCATGGCTACAGAGAACTAACTATACCACCAGCTAACAACTTCAAGATGGAATCTAATCATTTGCACATCTGGCCGAGGGGTGAGTTCATGTTGATTGCGCTTCCTAATCAAGACGACAGCTGGACTGTCACTCTTTTCATGCCTTTGGTTAAATTCGATGAATTACAAGGCAAAGAACATGCCATCAAGTCATTCTTTCAACAGAACTTTCCCGATGCTCTTCAATTAATCGGAGATTCAAAAGTGATCGAGGATTTCTCAAAACCATCTCATTTAGTTTCTATTAAGTGTACCAAATATAATGTAGGAAATAAAGCTGTAATACTAGGAGATTCAGCACATGCAATGGTACCCTTCTATGGCCAGGGGATGAATGCAGGTTTCGAAGACTGCCGCATTCTGGATGAATTCATACATAGCGAGGGCTTGGATTTAGAAGAGGCCCTAGAGTTATTTTCAATAACTAGAGCGAAAAATGCGGAAGCTATCTGCAACTTATCCATGTATAATTATAGTGAGATGAGGCAACTGGTCACGCAAACGTCTTATATCCTTCGGAAAAAGTTAGATAATATCCTTTATTGGCTTTTCTCAAGTAAATGGATTCCACTGTACCAGTCTATTACTTTTACTAACATACCCTACTCGGAATGTCTACAGAACAGAAAACGTCAAAATGTGATACTTCGAAATGTCTTTTATGTTCTTCTTTGTGCAAGTGTTACGCCCATTTCATGGTATATATTCAGGTGGAAACAATCTCTTTAA | XP_014286004.1\|kynurenine 3-monooxygenase, (78%; 86.06%) |
| ***β-Actin*** | ATGTGTGACGAAGAAGTTGCTGCTTTAGTTGTAGACAATGGATCCGGTATGTGCAAAGCCGGTTTTGCTGGAGATGATGCACCCCGAGCAGTATTCCCATCAATTGTTGGCAGGCCAAGGCATCAGGGTGTCATGGTTGGTATGGGCCAAAAGGACAGCTACGTAGGAGATGAAGCCCAAAGCAAAAGAGGTATCCTCACCCTGAAGTACCCCATTGAACACGGTATCATCACAAACTGGGACGACATGGAAAAGATCTGGCATCACACCTTCTACAACGAGCTGCGAGTCGCTCCAGAGGAACACCCCATCCTCCTCACCGAGGCTCCCCTCAACCCTAAAGCCAACAGGGAGAAGATGACCCAGATCATGTTTGAGACCTTCAACACCCCAGCCATGTATGTCGCCATCCAGGCTGTACTCTCCCTCTATGCCTCCGGTCGTACCACCGGTATTGTACTTGACTCAGGAGATGGTGTATCCCACACCGTACCCATCTATGAAGGTTATGCCCTTCCTCACGCCATCCTCCGTCTGGATCTTGCTGGACGTGACTTGACTGACTACCTTATGAAGATCCTCACCGAACGTGGTTACAGTTTCACAACCACCGCTGAAAGGGAAATCGTCAGGGACATCAAGGAAAAACTGTGCTATGTTGCCCTGGACTTTGAACAGGAAATGGCCACCGCTGCTGCCTCCACCTCCCTCGAGAAGTCTTATGAACTTCCTGACGGTCAGGTCATCACCATCGGTAACGAGAGGTTCCGTTGCCCAGAGGCTCTCTTCCAGCCTTCCTTCTTGGGTATGGAATCTTGCGGTATCCATGAGACTGTCTACAACTCCATCATGAAGTGCGATGTTGACATCAGGAAGGACTTGTACGCCAACACCGTCCTCTCCGGAGGTACCACCATGTACCCAGGTATTGCTGACAGGATGCAGAAGGAAATCACCGCCCTCGCTCCTTCAACCATCAAGATCAAGATCATCGCTCCCCCAGAAAGGAAGTACTCCGTATGGATCGGTGGTTCCATCTTGGCTTCCCTGTCCACCTTCCAGCAGATGTGGATCTCCAAGCAGGAATACGACGAATCCGGCCCAGGCATCGTCCACCGCAAATGCTTCTAA | XP_014279601.1\|actin, muscle, (99%, 99.47%) |
| ***α-Tubulin*** | ATGCGTGAGTGTATCTCCATACATGTTGGGCAAGCTGGCGTCCAGATTGGTAACGCCTGCTGGGAGCTGTACTGCTTGGAGCATGGGATCCAGCCTGACGGCCAGATGCCCTCAGACAAGACTGTTGGAGGAGGTGATGACAGCTTTAACACTTTCTTTTCCGAGACTGGTGCTGGTAAACACGTCCCAAGGGCTGTGTTTGTCGATCTTGAACCAACTGTTGTTGATGAGGTAAGGACCGGGACCTACAGGCAGCTTTTCCATCCAGAGCAGCTGATCACTGGCAAGGAAGATGCAGCAAACAACTATGCTCGTGGACACTACACCATTGGCAAAGAGATTGTCGACCTTGTACTGGACAGAATTCGTAAACTTGCCGATCAATGCACTGGTCTTCAGGGTTTCCTTATCTTCCACTCGTTCGGCGGAGGCACTGGTTCTGGATTTACATCTCTTCTCATGGAACGGTTGTCTGTTGACTATGGCAAGAAATCTAAACTGGAATTCGCCATCTACCCTGCTCCTCAGGTTTCCACTGCAGTAGTTGAACCATACAATTCCATCTTGACTACCCACACCACCCTTGAACACTCAGACTGTGCTTTTATGGTTGACAATGAGGCTATTTACGATATTTGCAGGCGAAATCTTGATATCGAACGACCCACTTATACCAATCTCAATAGGCTTATTGGACAAATTGTTTCATCTATCACAGCTTCTCTTCGGTTCGATGGTGCTTTGAATGTGGACTTGACTGAATTCCAGACCAACTTGGTACCTTATCCACGTATCCATTTCCCTCTGGTTACCTACGCCCCTGTGATCTCTGCCGAGAAAGCTTACCATGAACAACTCTCAGTAATGGAAATCACCAATGCTTGCTTTGAACCAGCCAACCAGATGGTGAAGTGTGATCCAAGGCATGGTAAATACATGGCCTGCTGCATGTTGTACAGGGGTGATGTCGTGCCCAAGGATGTAAATGCTGCTATTGCGACTATCAAAACCAAGAGAACAATTCAGTTTGTCGACTGGTGCCCGACAGGATTTAAGGTGGGCATCAACTACCAGCCCCCGACCGTCGTTCCTGGTGGTGACCTGGCCAAGGTCCAGAGAGCCGTGTGCATGCTGTCCAACACGACTGCTATCGCAGAGGCTTGGGCTCGCCTAGACCACAAGTTCGACCTCATGTACGCCAAGAGGGCCTTTGTCCATTGGTATGTTGGTGAAGGTATGGAGGAGGGAGAGTTCTCAGAGGCCAGGGAAGATTTGGCTGCCCTCGAGAAGGATTACGAAGAGGTCGGCATGGACTCAGTGGAAGGGGAGGGCGAAGGAGCAGAAGAGTACTGA | XP_014278495.1\|tubulin alpha-1 chain, (97%, 100%) |
| ***EF1-α*** | ATGCCCAAAGAAAAGATTCATATTAACATCGTCGTCATTGGACACGTAGATTCAGGTAAATCTACTACAACTGGCCATCTCATTTACAAATGTGGTGGTATCGATAAACGTACTATCGAAAAGTTCGAGAAAGAGGCTCAAGAGATGGGCAAAGGATCTTTCAAATATGCCTGGGTATTAGATAAGCTTAAAGCTGAACGTGAACGTGGTATCACAATCGATATTGCTTTGTGGAAATTCGAGACTGCTAAATACTATGTCACAATTATTGATGCCCCTGGTCACAGAGATTTCATCAAAAACATGATTACAGGGACATCACAGGCCGATTGTGCTGTGTTGATCGTTGCCGCAGGTACTGGTGAGTTTGAAGCTGGTATCTCTAAGAATGGTCAGACTCGTGAGCACGCACTTCTTGCCTTCACTCTTGGTGTAAAGCAATTGATTGTTGGAGTTAACAAAATGGACTCAACTGAACCAGCTTATTCAGAGCCCAGGTTCGAGGAAATCAAGAAAGAAGTATCGTCATACATCAAAAAGATTGGTTATAATCCAGCTTCTGTTGCCTTCGTTCCTATTTCTGGTTGGCATGGAGATAACATGCTTGAACCATCTGACAAGATGCCATGGTTCAAGGGGTGGGCTATTGAAAGGAAAGAAGGCAAAGCCGACGGTAAATGCCTTATTGAAGCCCTTGATGCCATCTTACCTCCTAGCAGGCCAACCGATAAGGCCCTTAGGCTTCCACTCCAGGACGTATACAAGATTGGTGGTATTGGAACAGTGCCAGTAGGACGAGTAGAGACTGGTTTATTGAAACCAGGTATGGTTGTCACTTTTGCTCCAGTCAATCTTACAACTGAAGTAAAGTCTGTTGAAATGCACCACGAAGCTCTCCAAGAAGCTGTTCCTGGTGATAATGTAGGTTTCAATGTCAAGAACGTTTCGGTCAAAGAATTGCGACGAGGTTATGTTGCTGGTGACTCCAAAAACAATCCACCCAAGGCTGCTGCTGACTTCACTGCCCAGGTCATCGTTTTAAATCATCCTGGTCAGATTTCCAATGGATATACTCCTGTACTTGACTGTCACACCGCCCATATTGCCTGCAAATTCGCCGAGATCAAAGAAAAGTGCGACCGTCGTACTGGTAAATCTACTGAAAGCAACCCAAAATCCATCAAGTCTGGTGATGCTGCCATCATCAACTTGGTCCCAACCAAACCAATGTGCGTAGAATCATTCCAGGAGTTCCCACCATTAGGTCGTTTTGCTGTAAGAGACATGAGGCAAACTGTTGCTGTCGGCGTCATCAAGTCGGTAATCAACAAAGATATAACAACGGGTAAAGTAACAAAGGCCGCCGAGAAGGCACAGAAGAAGAAATAA | XP_014270225.1\|elongation factor 1-alpha, (96%; 98.66%) |
| ***GAPDH*** | ATGTCTAAAATTGGTATTAATGGATTTGGAAGAATTGGGCGTCTTGTTCTTCGTAATGCTGTCGATAGTGGTGCCCAGGTAGTTGCTGTTAATGACCCCTTCATTAATGTGGAATATATGGTCTACCTTTTTAAATATGATTCCACACATGGACGTTTCAAGGGAAGTGTCTCATATTCTGGACAAAACCTTGTTGTGAATGGAAAAGAAATCCGTGTCTTCCAGGAAAGAGACCCAGCTAGCATTCCATGGGGTGAAGCCGGTGCCGAATATGTTGTTGAAGCTACTGGAGTTTTCACAACTGTTGAAAAGTGCAAGCCTCATTTGAAGGGTGGTGCCAAGAAAGTTATTATTACTGCACCTTCGGCTGATGCTCCCATGTTCGTTTGTGGTGTTAACTTGGATGCATACAAGCCAGATATGGACATAGTTTCTAATGCATCTTGCACAACTAACTGCCTTGCTCCATTAGCCAAAGTTATCCATGATAATTTTGGAATTGAAGAAGGTCTCATGACAACCGTTCATGCTGTTACTGCCACTCAGAAAACAGTTGATGGCCCATCTGGAAAGTTGTGGAGAGATGGAAGAGGTGCTGCTCAGAATATAATTCCAGCATCTACTGGTGCAGCTAAGGCTGTCGGCAAAGTCATTCCATCCCTAAATGGTAAACTGACTGGCATGGCTTTTAGAGTTCCTGTATCCAATGTTTCTGTTGTTGATTTAACCGCAAGATTGCAGAAGGATGCTACTTATGAAGAAATTAAAAAGAAAATTAAGGAAGCTTCAGAAGGTCCCCTAAAAGGAATTTTGGCATATACTGATGAACAGGTGGTCTCTTCCGATTTCATCGGCAATACTGCTTCATCTACTTTCGATGCTGAGGCTGGTATCCAACTGAACAACAAATTTGTAAAGCTCATCTCATGGTACGATAATGAATGTGGTTATTCCATGAGAGTCGTTGATCTTATCAGATACATGCAAACAAAGGATTAG | XP_014281222.1\|glyceraldehyde-3-phosphate dehydrogenase 2-like isoform X1, (99%, 98.19%) |

**Table S2.** Primer sequences and correspondent RT-qPCR parameters for the SGSB.

| **Gene ortholog** | **Primer sequence 5’-3’** | **Product (bp)** | **Efficiency (%)** | **R^2^** |
| --- | --- | --- | --- | --- |
| ***white*** | F: CTTTGGCAGAGGTACCAGTATT | 90 | 99.3 | 0.9989 |
|  | R: TGACGAACGCAGGGTTAAG |  |  |  |
| ***vermilion*** | F: CTGGTGGATCTTCCGGTTATC | 126 | 92.2 | 0.9932 |
|  | R: GACTGCTCAATGGTGGTATGT |  |  |  |
| ***cinnabar*** | F: GAGCATGGCTACAGAGAACTAAC | 128 | 99.9 | 0.9932 |
|  | R: GTCCAGCTGTCGTCTTGATTAG |  |  |  |
| ***α-Tubulin*** | F: GGTGCTTTGAATGTGGACTTG | 82 | 96.6 | 0.9996 |
|  | R: CGTAGGTAACCAGAGGGAAATG |  |  |  |
| ***β-Actin*** | F: GATCTGGCATCACACCTTCTAC | 106 | 97.2 | 0.9998 |
|  | R: CATCTTCTCCCTGTTGGCTTTA |  |  |  |
| ***EF1-α*** | F: CCAGGTTCGAGGAAATCAAGAA | 103 | 97.0 | 0.9998 |
|  | R: TCTCCATGCCAACCAGAAATAG |  |  |  |
| ***GADPH*** | F: GGTGCCGAATATGTTGTTGAAG | 109 | 94.4 | 0.9992 |
|  | R: CAGCCGAAGGTGCAGTAATA |  |  |  |

**Table S3.** Ranking of SGSB candidate reference genes under dsRNA treatments and their correspondent stability parameters.

| **Rank** | ***ΔCt*** | | ***BestKeeper*** | | ***NormFinder*** | | ***GeNorm*** | |
| --- | --- | --- | --- | --- | --- | --- | --- | --- |
|  | **Gene** | **SD^a^** | **Gene** | **SD^b^** | **Gene** | **Stability** | **Gene** | **M value** |
| 1 | α-Tubulin | 1.68 | α-Tubulin | 0.22 | α-Tubulin | 0.17 | β-Actin/ α-Tubulin | 0.34 |
| 2 | β-Actin | 1.72 | β-Actin | 0.28 | EF1-α | 0.39 |  |  |
| 3 | EF1-α | 1.77 | EF1-α | 0.51 | β-Actin | 0.55 | EF1-α | 0.50 |
| 4 | GAPDH | 4.18 | GAPDH | 4.06 | GAPDH | 4.16 | GAPDH | 2.34 |

^a^ Mean standard deviation given for the variation in Ct values

^b^ Standard deviation of the crossing point

**
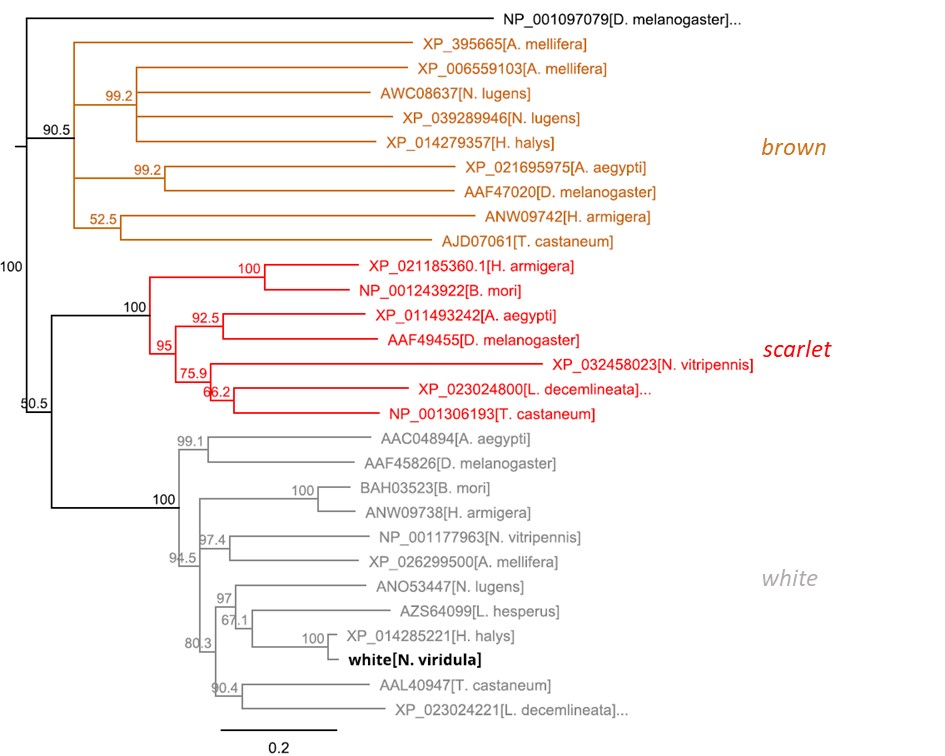
**

**Figure S1.** Phylogenetic tree constructed for white, scarlet and brown amino acid sequences that have been previously annotated in genomic studies and/or were found in BLASTp results for the SGSB sequence targeted in this study. Amino acid sequences were aligned using MUSCLE 3.8.425 with maximum of 8 iterations followed by tree construction using Jukes-Cantor genetic distance model and Neighbor-Joining method in Geneious prime 2022.1.1. Numbers in the tree indicate consensus support percentage (1000 bootstrap replicates). The amino acid sequence of an ABC transporter expressed in trachea, isoform D of *Drosophila melanogaster* (Accession: NP_001097079) was chosen as an outgroup. The phylogenetic construction confirmed the identity of the SGSB target sequence as *white* (in bold).

**
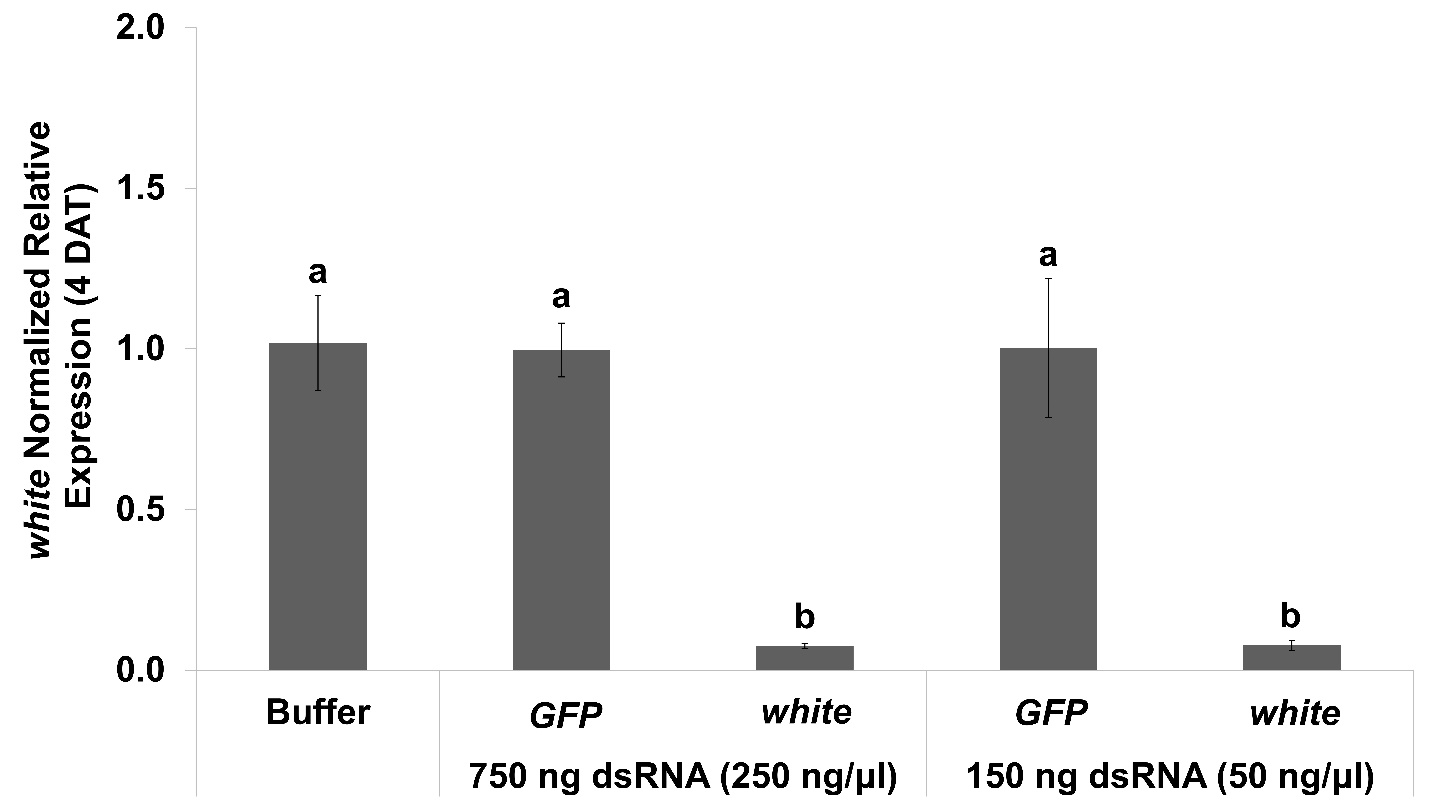
**

**Figure S2.** Sensitivity of newly emerged adult SGSB females to RNAi-based microinjection method targeting the *white* gene. Graphs show *white* normalized relative expression means ± SE. Significant *white* knockdown ($F_{\left( 4,25 \right)}=35.37;p<0.0001)$ was observed at four days after treatment (4DAT) using either 150 or 750 ng *white* dsRNA relative to *GFP* dsRNA- and elution buffer-treated females. Treatment means followed by the same letter were not statistically different (Fisher’s LSD test, α=0.05).


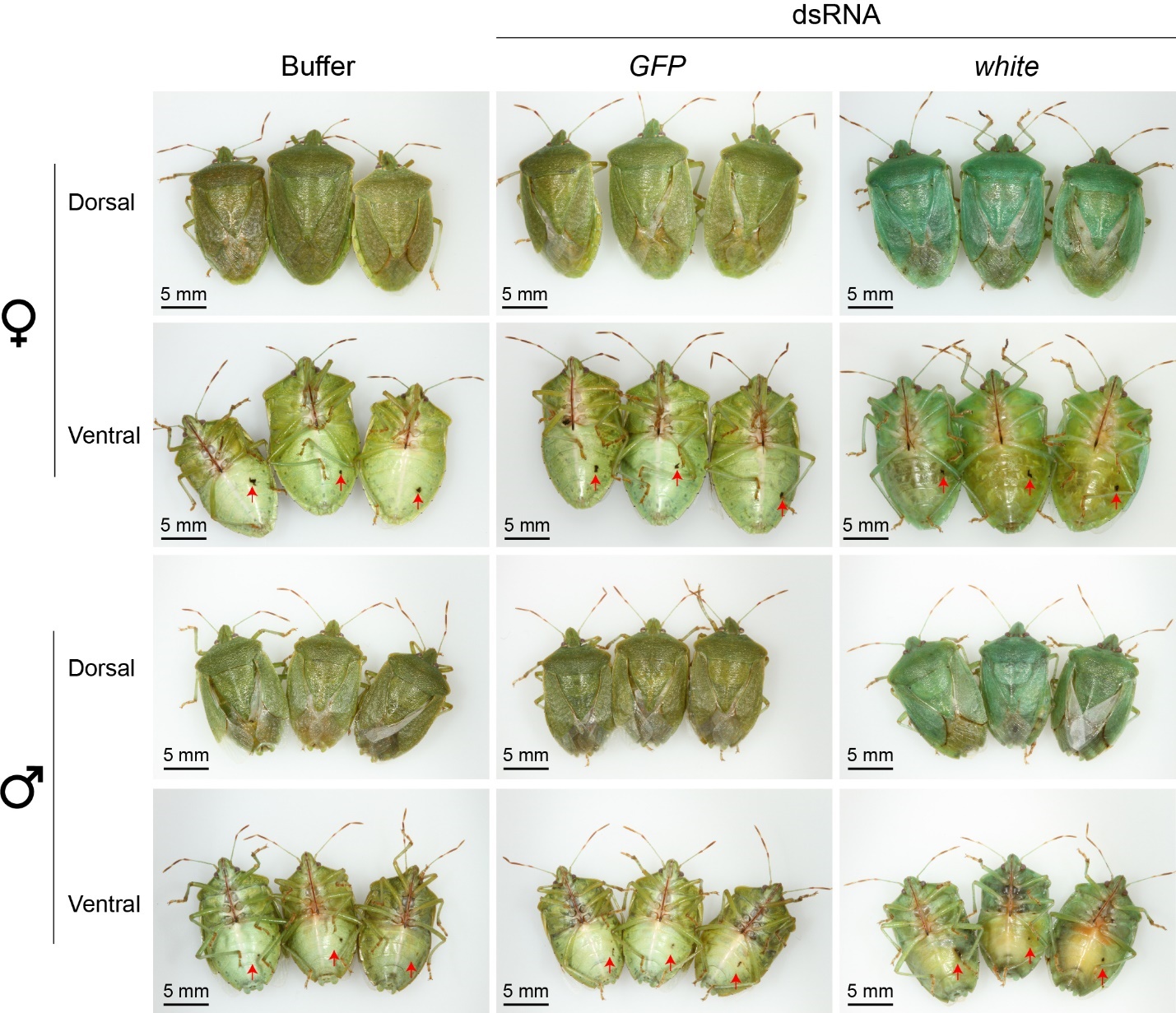


**Figure S3.** Phenotypes observed in 30 days-old SGSB adults following RNAi-based microinjections performed at <24 h after emergence. Columns show the phenotype across treatments (buffer and *GFP* dsRNA controls, and *white* dsRNA). Lines show dorsal and ventral views of females and males from each treatment. Red arrows show the scar where the microinjections were performed in newly emerged SGSB adults.

**
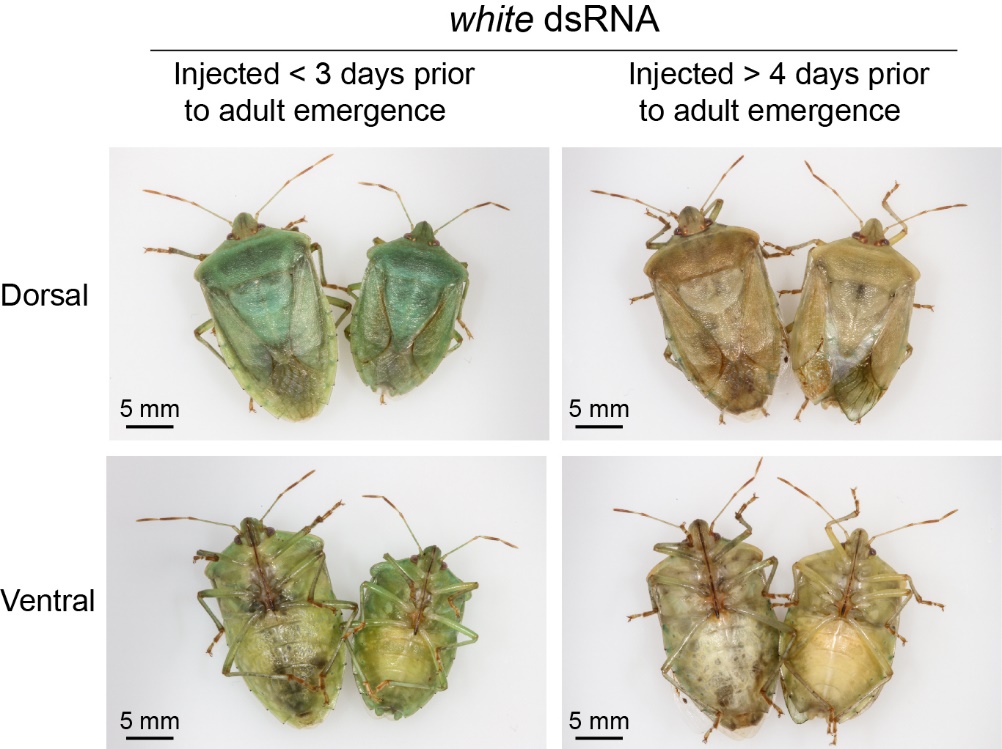
**

**Figure S4.** *white* knockdown phenotypes observed in 30 days-old SGSB adults following RNAi-based microinjections performed during the fifth instar nymphal stage at either <3 days prior emergence or >4 days prior to emergence.

**
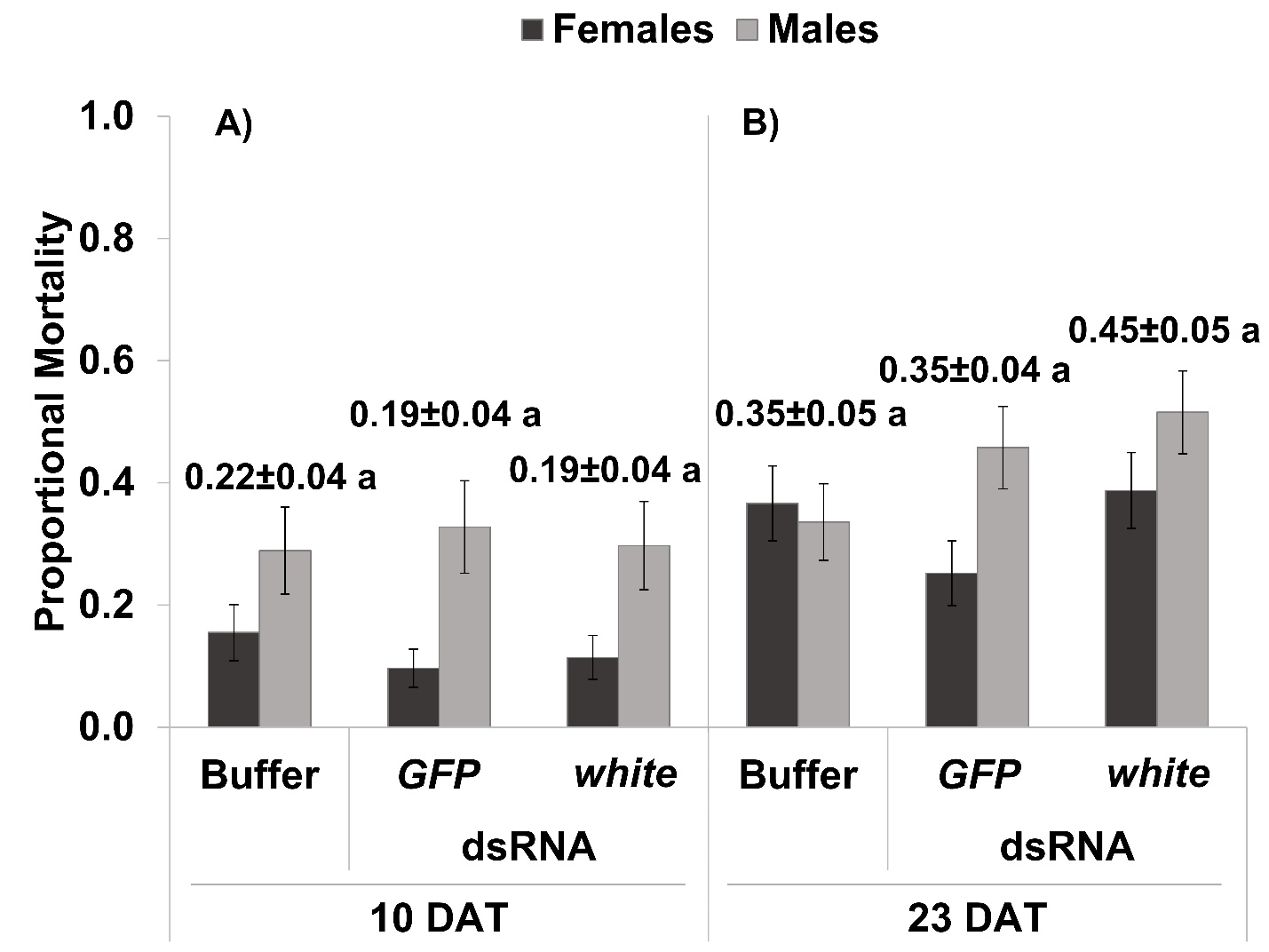
**

**Figure S5.** Mortality of adult SGSB following RNAi-based microinjections by **A)** ten $(F_{\left( 2,53 \right)}=0.17;p=0.8421)$ and **B)** 23 $(F_{\left( 2,51 \right)}=0.57;p=0.5667)$ days after treatment (DAT). Bars discriminate the proportional mean mortality ± SE of males and females, and above bars show the overall adult proportional mean mortality ± SE for each treatment. Within time-point, treatment means followed by the same letter were not statistically different (Fisher’s LSD Test, α=0.05).

**
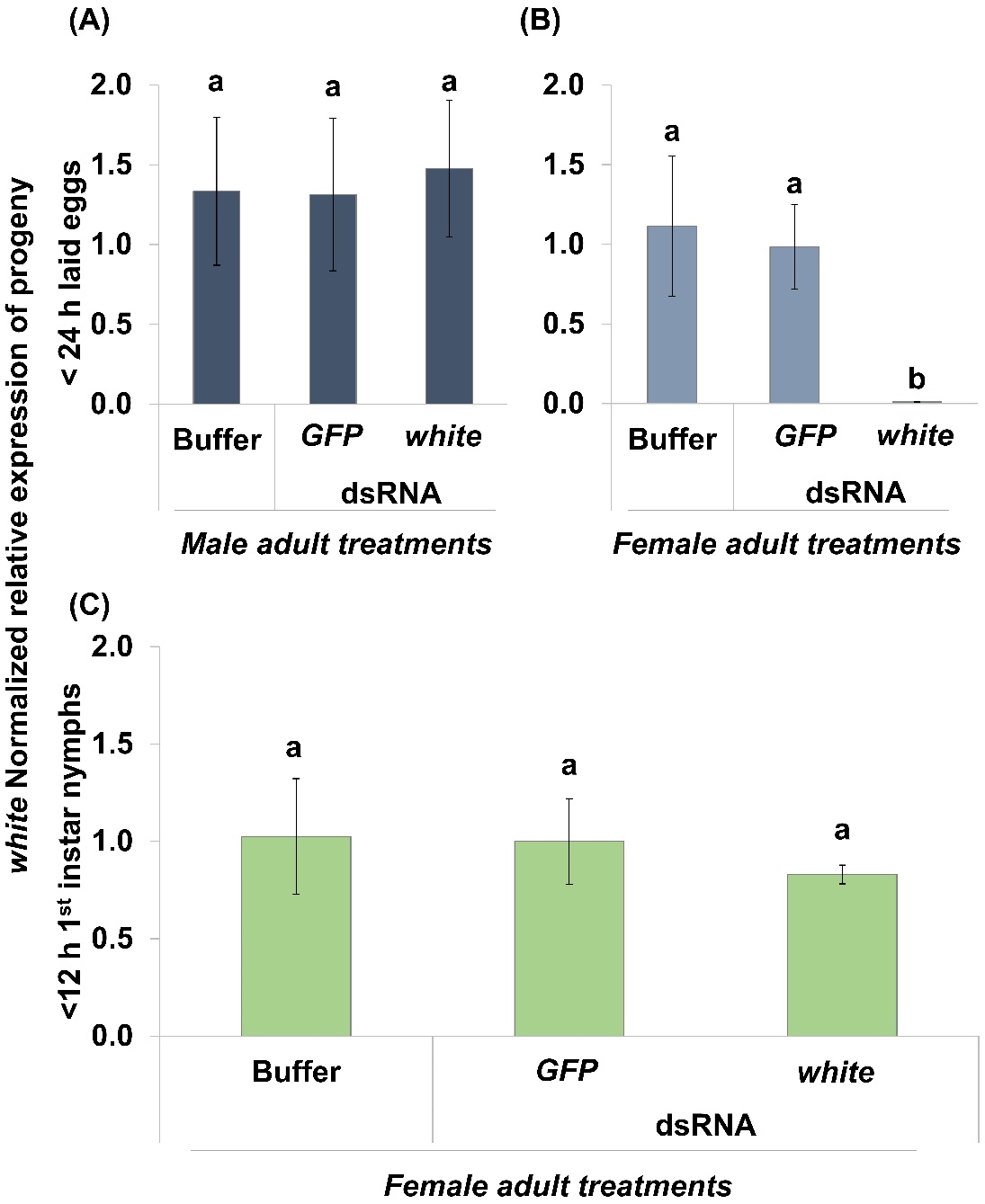
**

**Figure S6.** Parental RNAi effect on mean *white* expression ± SE in eggs and first instar nymphs of SGSB where adults treated with 150ng of *white*/*GFP* dsRNA and buffer-only were paired with non-treated adults. Relative to control groups, *white* gene expression was **A)** not significantly different in eggs generated from the mating of *white* dsRNA treated males $(F_{\left( 2,6 \right)}=0.05;p=0.9558)$; **B)** significantly different in eggs generated from *white* dsRNA treated females $(F_{\left( 2,6 \right)}=9.1;p=0.0152)$; and **C)** no longer significantly different in first instar nymphs of *white* dsRNA treated females $(F_{\left( 2,9 \right)}=0.54;p=0.5999)$. Treatment means followed by the same letter were not statistically different (Fisher’s LSD test, α=0.05).

**
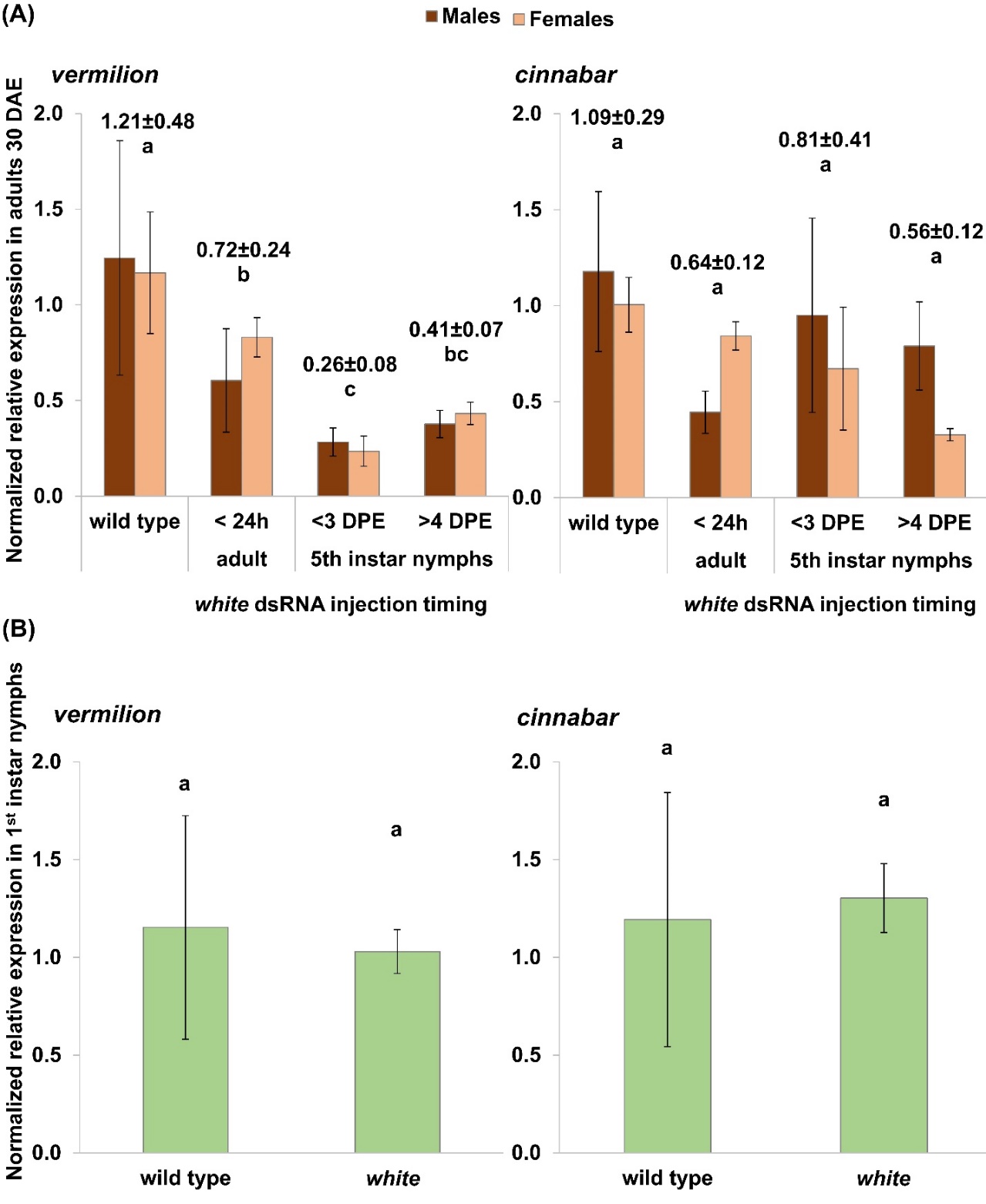
**

**Figure S7.** Mean relative expression of *vermilion* and *cinnabar* ± SE in the SGSB following RNAi-based experiments targeting the *white* gene. **A)** SGSB adult males and females at 30 days after emergence that were treated with 150 ng of *white* dsRNA as <24h newly emerged adults or as fifth instar nymphs either at <3 days prior adult emergence (DPE) or >4 DPE. Bars ± SE discriminate expression by sex and above bars show the overall adult mean expression ± SE for each treatment. Relative to wild type control, the expression of *vermilion* was significantly different in *white* dsRNA-treated SGSB males and females $(F_{\left( 3,28 \right)}=10.08;p<0.0001)$, while the expression of *cinnabar* was not significantly different $(F_{\left( 3,28 \right)}=1.67;p=0.1959)$. **B)** First instar nymphs from females treated at <24h after adult emergence. The expression of *vermilion* ($t_{\left( 4 \right)}=0.3;p=0.7813$) and *cinnabar* ($t_{\left( 4 \right)}=0.23;p=0.8283$) were not significantly different in nymphs from *white* dsRNA-treated females relative to wild type control. Treatment means followed by the same letter were not statistically different (Fisher’s LSD test, α=0.05).
